# Ruminal-buccal microbiota transmission and their diagnostic roles in subacute rumen acidosis in dairy goats

**DOI:** 10.1186/s40104-025-01162-4

**Published:** 2025-03-02

**Authors:** Tao Liu, Jingyi Xu, Xiaodong Chen, Jianrong Ren, Jinhui He, Yue Wang, Yangchun Cao, Le Luo Guan, Junhu Yao, Shengru Wu

**Affiliations:** 1 College of Animal Science and Technology, Northwest A&F University, Yangling, Shaanxi 712100 China; 2 Key Laboratory of Livestock Biology, Northwest A&F University, Shaanxi, 712100 China; 3 College of Animal Science and Technology, Ningxia University, Yinchuan, 750021 China; 4 Faculty of Land and Food Systems, the University of British Columbia, Vancouver, BC V6T 1Z4 Canada; 5 Department of Agricultural, Food and Nutritional Science, University of Alberta, 116 St. and 85 Ave, Edmonton, AB T6G 2P5 Canada

**Keywords:** Dairy goats, Diagnosis, Oral microbiota, Ruminal microbiota, Subacute rumen acidosis

## Abstract

**Background:**

Subacute rumen acidosis (SARA) is a common metabolic disorder in ruminants that disrupts the rumen microbiome and animal health, but diagnosis is challenging due to subtle symptoms and invasive testing requirements. This study explores the potential of the buccal (oral) microbiome as a diagnostic indicator for SARA, hypothesizing an interaction with the rumen microbiome.

**Results:**

The study involved 47 dairy goats, including 11 on a control diet and 36 on high-concentrate diets with increasing rumen-degradable starch. Animals were grouped based on dietary exposure and ruminal pH: Control, Low-RDS Tolerance/SARA (LRDST/LRDSS), and High-RDS Tolerance/SARA (HRDST/HRDSS). Transcriptomics of rumen epithelium showed heightened inflammatory pathway gene expression in SARA-susceptible goats compared to controls and tolerant groups. Alpha diversity of ruminal bacteria showed lower Shannon diversity in HRDSS goats compared to HRDST whereas buccal bacteria displayed significantly lower Chao1 diversity in LRDSS goats compared to HRDST. Beta diversity analyses revealed distinct patterns between SARA-affected goats and healthy controls in both ruminal and buccal microbiomes. *Prevotellaceae_UCG-003* emerged as a candidate biomarker, with reduced abundance in SARA-susceptible goats in both rumen and buccal samples. Machine learning classifiers achieved high accuracy in distinguishing SARA-susceptible goats using this genus (rumen AUC = 0.807; buccal AUC = 0.779). Source tracking analysis illustrated diminished cross-population of bacteria from the buccal to rumen (2.86% to 0.25%) and vice versa (8.59% to 1.17%), signifying compromised microbial interchange in SARA-affected goats. A microbiota transplant experiment verified SARA microbiota's ability to induce pH decline, escalate inflammation-related gene expression (*MAPK10*, *IL17B*, *FOSB*, *SPP1*), disrupt microbial transfer, and reduce *Prevotellaceae_UCG-003* in recipients.

**Conclusion:**

Our findings highlight SARA’s dual impact on ruminal and buccal microbiota, exacerbating epithelial inflammation gene expression. Shifts in the buccal microbiome, specifically reductions in *Prevotellaceae_UCG-003*, mirror ruminal changes and can be influenced by inter-compartmental bacterial transmission, thereby offering a non-invasive diagnostic approach for SARA.

**Supplementary Information:**

The online version contains supplementary material available at 10.1186/s40104-025-01162-4.

## Introduction

Subacute rumen acidosis (SARA) is a digestive and metabolic disorder that often seriously jeopardizes the health and lactation performance of dairy ruminants when they are fed with high energy diet [1–4]. Generally, SARA is characterized by a low ruminal pH below 5.6 or 5.8 and lasts for more than 180 min/d [5–7]. However, the diagnosis of SARA is difficult, as clinical symptoms are imperceptible and usually delayed from the time of incidence. Past research has suggested that ruminal microbiota could be a potential biomarker to help predict SARA due to its direct responses to the high grain and/or high energy diets [8–10]. It has been shown that when ruminants developed SARA, the abundance of Gram-negative bacteria and cellulolytic bacteria decreased in the rumen [11], which can lead to reduced fiber degradation capacity, decreased volatile fatty acids (VFAs) production, and reduced intake [12, 13]. However, the assessment of rumen microbiota under a commercial production setting is not feasible, and the collection of ruminal fluid uses the methods such as rumenocentesis and oral stomach tube which can be invasive and cause animal stress [14, 15]. Further, the difference in individualized susceptibility to SARA under high energy diet raised necessity to identify the novel way to identify SARA in dairy ruminants [5, 16, 17].

Recently, the oral microbiota has been shown to consist of several different microbial compositions residing differing habitats in teeth, buccal cavity, etc. [18]. It has also been shown that oral microbiota communicates with the gastrointestinal microbiota [19] and is closely related to the occurrence of several gastrointestinal diseases such as inflammatory bowel disease [20–22]. In Ruminants, the rumination is a behavior that ruminal substances regurgitation back into the mouth [23], suggesting that the rumen microbiota may play a role in affecting oral (including the teeth and buccal cavity) microbiota or vice versa [24]. Several studies have compared oral and rumen samples of ruminants under different feeding strategies (high or low grain feeding), different age and time of weaning, and showed that the oral microbiota can reflect fluctuations in the rumen microbiota in a timely manner [16, 24–26]. Therefore, because SARA occurrence is accompanied with changes in the rumen microbiota [8–10, 12, 13], we speculate that the oral (buccal) microbiome can interact with the rumen microbiome and serve as predictive markers of rumen bacteria-tissue interactions.

Due to the lack of rapid and effective diagnosis tools, it is difficult to evaluate host impaired functions that can affect animals’ health and welfare under SARA. Base on the rumination characteristic of ruminants and the rumen microbial role in identifying SARA occurrence. We further speculated that shifted oral microbiota under SARA may also be associated with the SARA occurrence and rumen tissue functional changes. Therefore, in this study, we aimed to address the mechanism of interaction between oral and rumen microbiota to regulate SARA and rumen epithelial inflammation to find oral microbial indicators for the diagnosis of SARA. This study offers a theoretical foundation and practical recommendations for the microbial mechanism of SARA diagnosis.

## Material and methods

### Ethics approval statement

This experiment was conducted at the Animal Research and Technology Centre of Northwest A&F University (Yangling, Shaanxi, China) in accordance with the recommended guidelines from the Administration of Affairs Concerning Experimental Animals (Ministry of Science and Technology, China, revised 2004).

### Animals and study design

Forty-seven healthy, multiparous dairy goats with ruminal fistulas and a similar average body weight of ~ 40 kg, average age 2–3 years in non-lactation period were used in this experiment. Two high-concentrate diets with gradually increasing amounts of rumen-degradable starch were designed to induce SARA in ruminal cannulated dairy goats. Briefly, 11 dairy goats that were randomly selected as the control (CON) group were fed a basal diet in 30% concentrate, dry matter (DM) basis (Additional Table S1). The other 36 dairy goats were subsequently fed a high-concentrate diet supplemented with whole corn (70% concentrate, DM basis, also referred to as the low-rumen-degradable-starch diet) (Additional Table S1). A total of 2 kg of total mixed ration (TMR) experimental diet was fed to each goat twice daily at 0800 h and 1700 h. The pH values of the ruminal fluids were measured every hour for 14 consecutive hours after feeding in the morning every day (details are shown in the sample collection section below). Approximately 100 mL of ruminal fluid was collected every hour for 14 consecutive hours after the morning feeding, after which the fluid was strained through 4 layers of sterile cheesecloth. The pH of the ruminal fluid was measured immediately with a mobile pH meter (HI 9024 C; HANNA Instruments, Woonsocket, RI, USA). When fed with the high-concentrate diets with whole corn, the goats exhibited SARA occurrence or healthy (SARA tolerance) conditions according to continuous identification of ruminal pH alterations. The dairy goats (*n* = 9), whose pH was lower than 5.8 for more than 3 h, were determined to be low-rumen-degradable-starch SARA (LRDSS) goats. The other dairy goats without reduced ruminal pH values were identified as low-rumen-degradable-starch tolerance (LRDST) goats. After 5 dairy goats were retained, the remaining dairy goats in the LRDST group were further fed a high-rumen-degradable-starch diet with crushed corn (70% concentrate, DM basis) (Additional Table S1). Similarly, according to the presence or absence of SARA, dairy goats were divided into high-rumen-degradable-starch SARA (HRDSS, *n* = 7) and high-rumen-degradable-starch tolerance (HRDST, *n* = 15) groups. The experiment lasted for 63 d including adaptation period for 7 d, and the experimental and data collection periods for each diet lasted for 28 d. Briefly, a basal diet for CON lasted 63 d, low level of RDS diet for LRDSS and LRDST groups lasted 56 d, high level for RDS diet for HRDSS and HRDST groups lasted 28 d. All the dairy goats in the experiment were fed in a single pen with free access to water and space for their activities.

### Sample collection of dairy goats

**Fig. 1:**
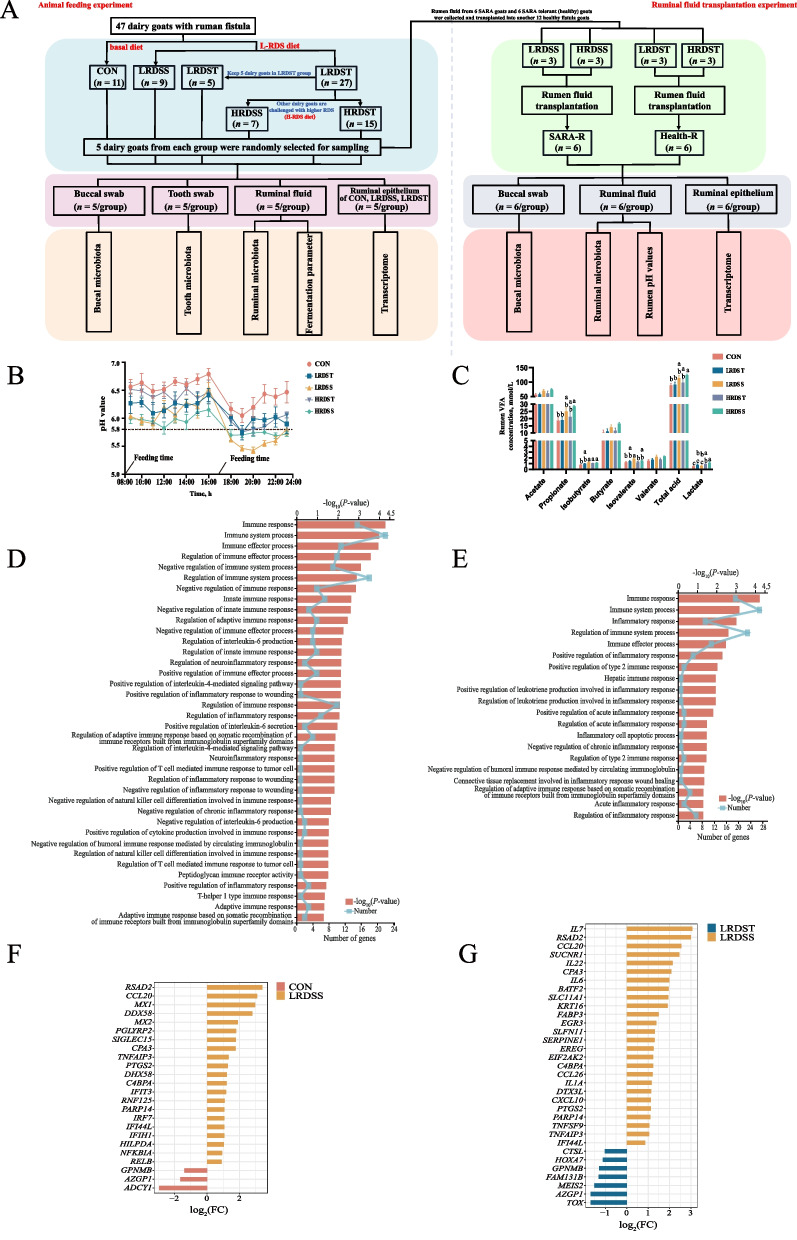
Identification of SARA occurrence and SARA tolerance in goats fed gradually increasing amounts of rumen-degradable starch. **A** Flow chart of animal feeding experiment and ruminal fluid transplantation experiment design, as well as the sample collection. **B** Changes in the rumen pH values of five groups (CON, LRDSS, LRDST, HRDSS, and HRDST) of dairy goats 14 h after morning feeding. **C** Comparison of rumen fermentation parameters among the CON, LRDST, LRDSS, HRDST and HRDSS groups. ^a−c^Mean values within an index with the same superscript letters indicate no significant difference. **D** and **E** The immune-related GO enrichment terms of the LRDSS vs. CON DEG dataset (**D**) or the LRDSS vs. LRDST DEG dataset (**E**). **F** and **G** The selected DEGs between the CON and LRDS groups (**F**) and between the LRDST and LRDS groups (**G**) that were enriched in immune-related GO enrichment terms

When the goats were divided into 5 different groups, CON, LRDSS, LRDST, HRDSS, and HRDST, 5 dairy goats from each group were randomly selected for sampling. Five goats in CON, LRDSS and LRDST whose pH was close to the average ruminal pH (presented in Fig. 1B) were selected as representative goats and were subsequently slaughtered 2 h after the morning feeding. Then, a total of 50 mL of strained ruminal fluid was collected and stored at −80 °C to analyse the changes in rumen microorganisms and rumen fermentation. Medical swabs were used to collect buccal and tooth microbiota samples at the same time. The swabs were gently rubbed against the buccal and tooth, moving in a circular motion to maximize contact with the mucosal epithelium and tooth, after which the swabs were quickly placed into collection tubes with DNA/RNA Shield (ZYMO, USA) and stored at −80 °C until laboratory preparation. Furthermore, approximately 1 cm^2^ of epithelial tissue from the dorsal rumen at a similar position was collected from each goat, stored in liquid nitrogen overnight and then stored at −80 °C for RNA isolation and transcriptome analysis [27].

### Ruminal microbiota transplantation (RMT) from donor SARA and healthy (SARA tolerance) goats to healthy recipient goats

Selecting another twelve healthy dairy goats with rumen fistulas, pre-fed a basal diet (30% concentrate, DM basis) (Additional Table S1). On the slaughter days, the ruminal fluid of the 6 donor SARA dairy goats in the LRDSS (*n* = 3) and HRDSS (*n* = 3) groups, as well as 6 SARA-tolerant (healthy) dairy goats in the LRDST (*n* = 3) and HRDST (*n* = 3) groups, were collected and then transplanted to another 12 healthy dairy goats after the ruminal fluid content was removed. Briefly, on the day of exchange, the rumen contents were first completely removed from all animals except those in the control group, after which the rumen was rinsed with 30 L of sterile prewarmed phosphate-buffered saline (PBS, pH 6.8) at least three times until the solution was colourless. Finally, the rumen contents were transferred. These 2 groups of recipient goats were subsequently named the SARA-R and Healthy-R groups. To avoid the adverse effects of oxygen exposure on the rumen microbiome, four people worked together for the rinsing and exchange procedure, ensuring that the whole process was processed within 15 min for each animal. The detailed steps were performed in accordance with the methods of Zhou et al. [28]. After transplantation, the goats were further fed a normal concentration of feed (50% concentrate, DM basis) for 2 weeks, after which their buccal swabs, ruminal fluid, and ruminal epithelial tissue were collected and stored in accordance with the above-described description in the “Sample collection of dairy goats” section.

### Determination of volatile fatty acids (VFAs) in ruminal fluid

Before the VFAs were measured, the standard materials (dissolved every 0.5 g in 1 mL of sterile water until completely dissolved) and ruminal fluid were centrifuged at 13,000 × *g* for 10 min and subsequently supernatant was analysed as previously described [29]. In brief, 4 mL of each supernatant was mixed with 1 mL of metaphosphoric acid (250 g/L) and then centrifuged for 15 min at 10,000 × *g* and 4 °C. Two milliliters of the supernatant was mixed with 200 µL of crotonic acid (10 g/L) and then filtered through a 0.45-µm filter. The VFAs were separated and quantified with an Agilent 7820A GC system equipped with a polar capillary column (AE-FFAP, 30 m × 0.25 mm × 0.33 μm) and a flame ionization detector (FID).

### Determination of the concentration of lipopolysaccharide (LPS) and lactate in ruminal fluid

The detailed procedures for sample preparation, LPS determination, and lactate measurement have been described previously [6, 30]. Briefly, the ruminal fluid was boiled at 100 °C for 30 min, after which the limulus amoebocyte lysate (LAL) assay was used for LPS determination. The assay was performed using a 96-well microplate kit, and the absorbance was read at 405 nm on a microplate reader (model 3550; Bio-Rad, Hercules, CA, USA). Then, 550 µL of ruminal fluid was mixed with 200 µL of 20% perchloric acid, shaken for 60 s, and incubated for 10 min on ice. The mixture was subsequently centrifuged for 20 min at 14,000 × *g* at 4 °C to collect the supernatant. The supernatant was centrifuged again for 20 min at 14,000 × *g* at 4 °C before being filtered through a 0.22-µm filter. Lactate was quantified with an Agilent 7820A GC system equipped with a polar capillary column (AE-FFAP, 30 m × 0.25 mm × 0.33 μm) and a FID.

### DNA extraction

Total microbial DNA was extracted from ruminal fluid and oral swabs according to the instructions of the E.Z.N.A.® Soil DNA Kit (Omega Bio-Tek, Norcross, GA, USA). Unused swabs and nuclease-free water were used as negative controls for DNA extraction in the same way. The quality and concentration of the extracted DNA were determined by 1.0% agarose gel electrophoresis and a NanoDrop® ND-2000 spectrophotometer (Thermo Scientific, USA). The total DNA was stored at −80 °C until further use.

### Bacterial 16S rRNA gene amplicon sequencing and data analysis

The hypervariable region V3–V4 of the bacterial 16S rRNA gene was amplified by PCR using the forward primer 338F (5′-ACTCCTACGGGAGGCAGCAG-3′) and the reverse primer 806R (5′-GGACTACHVGGGTWTCTAAT-3′) [31]. For library preparation, DNA-free water and the ZymoBIOMICS Microbial Community DNA Standard (Cat. No. D6305, Zymo Research, Irvine, USA) were included as negative and positive PCR controls, respectively. The negative and positive controls from the extraction phase were also subjected to library preparation and sequencing. The procedure was as follows: predenaturation at 95 °C for 3 min; 27 cycles of denaturation at 95 °C for 30 s, annealing at 55 °C for 30 s, and extension at 72 °C for 30 s; stable extension at 72 °C for 10 min; and stored at 4 °C (PCR thermocycler: ABI GeneAmp® 9700). The PCR mixture included 4 μL of 5 × TransStart FastPfu buffer, 2 μL of 2.5 mmol/L dNTPs, 0.8 μL of forward primer (5 μmol/L), 0.8 μL of reverse primer (5 μmol/L), 0.4 μL of TransStart FastPfu DNA polymerase, 10 ng of template DNA and ddH_2_O to a final volume of 20 µL. Each sample was amplified in triplicate. PCR products from the same sample were mixed and resolved via 2% agarose gel electrophoresis. The recovered products were purified with an AxyPrep DNA Gel Extraction Kit (Axygen Biosciences, Union City, CA, USA) and quantified with a Quantus™ fluorometer (Promega, USA). A library of purified PCR products was established using the NEXTFLEX Rapid DNA-Seq Kit and sequenced on the NovaSeq PE250 platform (Illumina, San Diego, CA, USA).

The raw sequences were merged with FLASH (v1.2.11) [32] and quality filtered with fastp (0.19.6) [33, 34]. The sequences were imported into QIIME2 v2021.8 for demultiplexing and construction of an amplicon sequence variant (ASV) table using DADA2 [35, 36]. Bacterial 16S ASVs were assigned a taxonomy using the SILVA database (version 138) as the reference. The relative abundance of a taxon in the sample was the fraction of the taxon observed in the ASV table relative to the sum of all observed taxa corresponding to the sample in the ASV table. Alpha diversity indices, including the Sobs, ACE, Chao1 richness estimate, Shannon diversity index and Phylogenetic diversity, were calculated using QIIME2 and analysed at the ASV level [36]. The differences in α diversity between groups were analysed by the Wilcoxon rank sum test. The similarity among the microbial communities in different samples was determined by principal coordinate analysis (PCoA) based on Bray–Curtis dissimilarity, and the PERMANOVA test was used to assess the percentage of variation explained by the treatment along with its statistical significance using the vegan v2.5–3 package. Taxa in ASV level with a relative abundance ≥ 0.1% were subjected to downstream analysis of correlation and comparison. The Wilcoxon rank sum test was performed to identify the genera with significant differences in relative abundance between different groups, and multiple test correction was performed by false discovery rate (FDR) analysis. Furthermore, partitioning around medoids (PAM) clustering was performed based on the Jensen‒Shannon divergence (JSD). The best clustering K number was calculated using the Calinski‒Harabasz (CH) index and further used to distinguish the microbiota types [37]. Co-occurrence networks were constructed to explore the internal community relationships across the samples [38]. A correlation between two nodes was considered to be statistically robust if the Spearman correlation coefficient was greater than 0.6 or less than −0.6 and the *P* value was less than 0.05. Moreover, the degree centrality, closeness centrality, and betweenness centrality were calculated to identify the key position genera.

### Random forest and support vector machine (SVM) analyses

The random forest package in R was used for the random forest analysis [39], with the identified oral and rumen bacteria being used as the inputs of the random forest model to classify SARA with the CON group. To further minimize potential overfitting in the model, an AUC-validation approach was applied. The number of trees in the forest was set to 500. The SVM model was used to select the features that contributed the most to the group difference. In the SVM classification analysis, we used algorithms implemented in Python from the sklearn open-source library for machine learning. Furthermore, the area under the curve (AUC) of the receiver operating characteristic (ROC) curve was calculated to analyse the sensitivity and specificity of the diagnostic power of the identified rumen and oral differential bacteria based on the 16S rRNA gene sequencing data for bacterial DNA abundance.

### Source tracker analysis of ruminal and oral microbiota

SourceTracker is a Bayesian approach used to predict the composition ratio of target samples from each source sample according to the microbial community structure distribution of the target samples and the source samples [40]. We employed SourceTracker to assess the contributions to the oral microbiota. We evaluated each subject’s buccal and teeth microbiota separately and provided only the subject’s own ruminal microbiota as potential sources. Source Tracker R script was downloaded from https://github.com/danknights/sourcetracker.

### RNA extraction, transcriptome sequencing and transcriptomics data processing

Fifteen rumen epithelial samples from the CON, LRDST, and LRDSS groups as well as the 12 rumen epithelial samples of recipient goats from the SARA-R and Healthy-R groups were measured for transcriptome analysis. Total RNA was extracted from the tissue using TRIzol® Reagent according to the manufacturer’s instructions (Invitrogen), and genomic DNA was removed using DNase I (TaKaRa). Then, RNA quality was determined by a 2100 Bioanalyzer (Agilent) and quantified using an ND-2000 (NanoDrop Technologies). Only high-quality RNA samples (OD_260/280_ ≥ 1.8, OD_260/230_ ≥ 1.0, RIN ≥ 6.5, 28S:18S ≥ 1.0, > 1 μg) were used to construct the sequencing library. All samples met analyzable criteria, with RIN values ranging from 7.4 to 10.0. The RNA-seq transcriptome library was prepared following the instructions of the TruSeq^TM^ RNA sample preparation kit from Illumina (San Diego, CA, USA) using 1 μg of total RNA. Then, a paired-end RNA-seq library was sequenced. The total RNA was subjected to transcriptome analysis via RNA-seq with 2 × 150 bp paired-end sequencing (PE150) on an Illumina HiSeq X Ten (2 × 150 bp read length).

The raw paired-end reads were trimmed and quality controlled by SeqPrep (https://github.com/jstjohn/SeqPrep) and Sickle (https://github.com/najoshi/sickle) with default parameters. Then, the clean reads were separately aligned to the reference genome in orientation mode using HISAT2 software [41] (http://ccb.jhu.edu/software/hisat2/index.shtml). The *Capra* *hircus* V1 (ARS1 [GCF_001704415.1]) gene annotation list was used as a reference genome. The mapped reads of each sample were assembled by StringTie (https://ccb.jhu.edu/software/stringtie/index.shtml?t=example) via a reference-based approach [42].

To identify differentially expressed genes (DEGs) between the two groups, the expression level of each transcript was calculated according to the fragments per kilobase per million reads (FPKM) method. RSEM [43] (http://deweylab.biostat.wisc.edu/rsem/) was used to quantify gene abundances. Essentially, differential expression analysis was performed using DESeq2 [44], and DEGs with |log_2_Fold Changes| > 1 and FDR value ≤ 0.05 were considered to be significant DEGs. In addition, GO functional enrichment analysis was performed to determine which DEGs were significantly enriched in GO or KEGG terms at a Benjamini-Hochberg (BH)-corrected *P* (FDR) value ≤ 0.05 compared with the whole-transcriptome background. GO functional enrichment was carried out by Goatools and KOBAS [45].

### Statistical analysis

After testing the normality and variance homogeneity of the data, the pH, VFA, lactate and LPS contents were statistically evaluated by one-way ANOVA using SPSS 21.0. In this study, correlations between variables were tested by the Spearman correlation test, with *P* < 0.05 and |*r*| > 0.6. To explore the correlation between rumen fermentation parameters and microbial communities, redundancy analysis (RDA) or canonical correspondence analysis (CCA) was performed in R. The length of the longest detrended correspondence analysis (DCA) ordination axis was 2.009, shorter than 3, indicating the applicability of RDA. A length longer than 4 was considered to indicate that the CCA was more suitable [46].

## Results

### Ruminal fermentation shifted and epithelial inflammation occurred in dairy goats with SARA

After continuously measuring rumen fluid pH for 14 h after feeding, it was below 5.8 for more than 6 h in the SARA-susceptible goats, including high rumen degradable starch-SARA group (HRDSS) and low rumen degradable starch-SARA group (LRDSS) (Fig. 1A and B). The average ruminal pH was below 5.8 for ~ 1 h in the low-RDS tolerance (LRDST) and high-RDS tolerance (HRDST) groups (Fig. 1A and B). Compared to those of the goats in the CON, the duration of rumen fluid pH values less than 5.8 was significantly greater in the LRDSS and HRDSS groups (Fig. 1A and B). The ruminal concentrations of propionate, isobutyrate, isovalerate and total VFAs were higher in SARA goats comparing to healthy goats (*P* < 0.05; Fig. 1C). For the VFAs molar ratio, there was no significant differences between SARA and healthy goats (*P* > 0.05; Additional Table S2). Furthermore, there were no significant differences in the concentrations of LPS between dairy goats with SARA and healthy dairy goats (*P* = 0.225), but the concentration of lactate was significantly higher in the HRDSS group than in the CON and LRDST groups (*P* < 0.001; Fig. 1C and Additional Table S2). Further, the comparison of rumen epithelium transcriptomes (Additional Fig. S1A), showed that 181 (110 upregulated and 71 downregulated) and 283 (124 upregulated and 159 downregulated) DEGs in the LRDSS group when separately compared with the CON group and the LRDST group (with criteria of |log_2_Fold Changes| ≥ 1 and FDR < 0.05), respectively (Additional Fig. S1B). Among those significantly differentially expressed genes, the enriched functions of inflammation related pathways were significantly higher in the LRDSS group compared with those in the CON and LRDST groups (*P* < 0.05; Fig. 1D–G).

### Rumen microbiota alteration and its association with rumen pH and fermentation changes during SARA occurrence

**Fig. 2:**
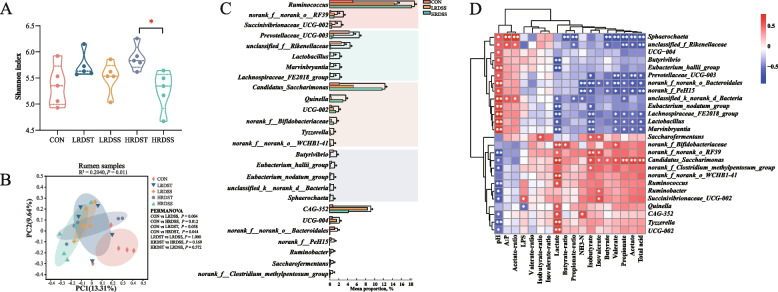
Comparison of microbiota in the rumens of dairy goats exhibiting SARA occurrence (susceptible) or healthy (SARA tolerance) status. **A** Comparison of ruminal microbial alpha diversity with the Shannon index among the CON, LRDSS, LRDST, HRDSS, and HRDST groups. **B** Comparison of ruminal microbial beta diversity among the CON, LRDSS, LRDST, HRDSS, and HRDST groups according to ANOSIM analysis based on the Bray–Curtis distance matrix. PERMANOVA was applied to analyse the microbial differences between the 2 groups. **C** Differential ruminal genera identified when comparisons among the CON group and SARA (LRDSS and HRDSS) groups were performed. The genera that significantly increased in both the LRDSS and HRDSS groups compared to the CON group are highlighted in red; the genera that significantly decreased in both the LRDSS and HRDSS groups compared to the CON group are highlighted in green; the genera that gradually increased in the CON LRDSS and HRDSS groups are highlighted in brown; and the genera that gradually decreased in the CON, LRDSS and HRDSS groups are highlighted in purple. The Mann–Whitney U test was carried out for the two groups. The Kruskal-Wallis test with the Tukey-Kramer post hoc test was employed for more than three groups. * indicates that the difference is significant at FDR < 0.05. **D** Spearman correlation between the common genus-level differences in the bacteria in dairy goats from the CON group and SARA (LRDSS and HRDSS) groups and their rumen fermentation parameters. * indicates that the difference is significant with *P* < 0.05, ** indicates that the difference is significant with *P* < 0.01

A total of 25 rumen samples were collected at the 2 h after morning feeding, obtaining 923,122 high-quality sequences from 22,235 ASVs (Fig. S2A and B). The α diversity analysis showed that HRDST dairy goats have more diversity of rumen microbiota compared to HRDSS goats via Shannon index (*P* < 0.05; Fig. 2A), while there were no significant differences in other α diversity index of ruminal microbiota between SARA and healthy (CON and SARA tolerance groups) dairy goats (*P* > 0.05; Additional Fig. S2C and D). However, β diversity of the rumen microbiota in the LRDSS and HRDSS groups were significantly separated from the CON group (*P*_(LRDSS vs.CON)_ = 0.004 and *P*_(HRDSS vs.CON)_ = 0.012) (Fig. 2B). Compared with those in the CON group, 26 genera were significantly differential abundant in the LRDSS and HRDSS groups. Compared with those in the CON group, the abundances of *Ruminococcus*, *norank_f__norank_o__RF39* and *Succinivibrionaceae_UCG-002* were significantly higher in both LRDSS and HRDSS groups (*P* < 0.05), and the abundances of *Prevotellaceae_UCG-003*, *unclassified_f__Rikenellaceae*, *Lactobacillus*, *Marvinbryantia* and *Lachnospiraceae_FE2018_group* were significantly lower in both LRDSS and HRDSS groups (*P* < 0.05; Fig. 2C). Moreover, compared with those in the CON group, the abundances of *Candidatus_Saccharimonas*, *Quinella*, *UCG-002*, *norank_f__Bifidobacteriaceae*, *Tyzzerella* and *norank_f__norank_o__WCHB1-41* were significantly higher in the HRDSS group (*P* < 0.05), while the abundances of *Butyrivibrio*, *Eubacterium_hallii_group*, *Eubacterium_nodatum_group*, *unclassified_k__norank_d__Bacteria* and *Sphaerochaeta* were significantly lower in the HRDSS group (*P* < 0.05). Next, the differential abundance analysis showed significantly higher relative abundances of *Quinella*, *norank_f__norank_o__Gastranaerophilales*, *norank_f__norank_o__WCHB1-41* and *unclassified_f__Selenomonadaceae*, as well as lower abundances of *Ruminobacter* in the LRDSS group when compared to the LRDST group (*P* < 0.05). And significantly higher relative abundances of *UCG-004*, *Anaeroplasma*, *norank_f__p-251-o5* and *Sphaerochaeta,* as well as significantly lower *Candidatus_Saccharimonas* were identified in the HRDSS group compared to the HRDST group (*P* < 0.05; Additional Fig. S2E and F). Moreover, the genera that increased in the LRDSS and HRDSS groups were significantly negatively correlated with the ruminal pH (rho ranged from −0.53 to −0.85, *P* < 0.05). The genera that were significantly more abundant in the CON group were significantly negatively correlated with the proportions of ruminal acetate, propionate, butyrate, isobutyrate, valerate, lactate and VFAs (rho ranged from −0.51 to −0.90, *P* < 0.05; Fig. 2D).

### Oral microbiota alteration and its association with ruminal pH and fermentation changes during SARA occurrence

**Fig. 3:**
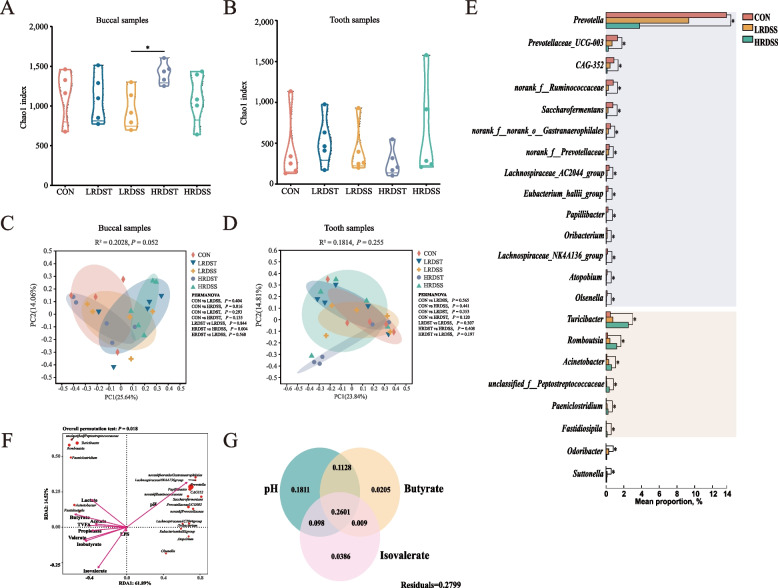
Comparison of oral (buccal and tooth) microbiota of dairy goats exhibiting SARA occurrence (SARA susceptible) or healthy (control and SARA tolerance) status. **A** and **B** Comparison of buccal and tooth microbial alpha diversity with the Chao1 index among the CON, LRDSS, LRDST, HRDSS, and HRDST groups. The Kruskal-Wallis test with the Tukey-Kramer post hoc test was employed to test microbial alpha diversity differences. * FDR < 0.05. **C** and **D** Comparison of buccal and tooth microbial beta diversity with ANOSIM analysis based on the Bray-Curtis distance matrix among the CON, LRDSS, LRDST, HRDSS, and HRDST groups. PERMANOVA was applied to analyse the microbial differences between the 2 groups. **E** Differential buccal genera identified when comparisons between the CON group and the SARA (LRDSS and HRDSS) groups were performed. The genera that gradually increased along the CON, LRDSS and HRDSS groups are highlighted in brown, and the genera that gradually decreased along the CON, LRDSS and HRDSS groups are highlighted in purple. The Mann–Whitney U test was carried out for the two groups. The Kruskal-Wallis test with Tukey-Kramer post hoc test was employed for more than three groups. * indicates that the difference is significant at FDR < 0.05. **F** Redundancy analysis (RDA) of differential buccal genera of the CON, LRDSS and HRDSS groups (red site names) and rumen fermentation parameters (pink arrows). The lengths of arrows indicate the magnitude of variance to which that variable could explain. Smaller angles between 2 variables indicate stronger correlations between these indices. **G** Variance partitioning analysis (VPA) revealed the effects of ruminal pH, butyrate and isovalerate on the buccal microbiota

A total of 25 buccal and tooth samples were collected, obtaining 1,035,398 high-quality sequences from 27,713 ASVs in buccal cavity, and 843,190 high-quality sequences from 11,251 ASVs in tooth (Fig. S3A–D). The α diversity analysis of the oral (buccal and tooth) microbiota showed a significantly lower buccal microbial Chao1 index was detected in the LRDSS group compared with the HRDST group (*P* < 0.05), but there was no significant differences in tooth microbial Chao1 index (*P* > 0.05; Fig. 3A and B). A significant difference in β diversity of the buccal microbiota was also detected between the HRDSS and CON groups (*P* = 0.0016) as well as between the HRDSS and HRDST groups (*P* = 0.004; Fig. 3C). However, no significant effect of SARA on β diversity of tooth microbiota was identified (*P* > 0.05; Fig. 3D). Further, the abundances of buccal *Prevotella*, *Prevotellaceae_UCG-003*, *CAG-352*, *norank_f__Ruminococcaceae*, *Saccharofermentans*, *norank_f__norank_o__Gastranaerophilales*, *norank_f__Prevotellaceae, Lachnospiraceae_AC2044_group, Papillibacter, Eubacterium_hallii_group, Lachnospiraceae_NK4A136_group, Atopobium, Oribacterium* and *Olsenella* gradually decreased in the CON, LRDSS group and the HRDSS group (*P* < 0.05; Fig. 3E). *Turicibacter, Romboutsia, Acinetobacter, unclassified_f__Peptostreptococcaceae, Paeniclostridium* and *Fastidiosipila* gradually increased in the CON group, the LRDSS group and the HRDSS group (*P* < 0.05; Fig. 3E). Furthermore, redundancy analysis (RDA) and variance partitioning analysis (VPA) revealed that the ruminal pH explained the largest proportion of the variation of buccal microbiota (65.18%), followed by the amounts of isovalerate (40.57%) and butyrate (40.24%) (Fig. 3F–G).

Furthermore, the significantly higher relative abundances of *Neisseria*, *Cornebacterium*, *Pseudomonas*, *Turicibacter*, and *Romboutsia,* as well as the significantly lower relative abundances of *NK4A214 group*, and *Succiniclasticum*, whose relative abundances were greater than 1%, were identified as significantly altered buccal genera in the LRDSS and HRDSS groups compared with the LRDST and HRDST groups (Additional Fig. S4A and B). Additionally, only the relative abundance of total *Bacteroides* of tooth samples, was greater than 1%, in the LRDSS and HRDSS groups significantly increased compared to that in the CON group (Additional Fig. S5A). Moreover, differentially abundant genera were identified in tooth microbial community between susceptible and tolerant dairy goats. Compared with the SARA-tolerant goats, the relative abundances of those low abundance tooth bacteria (relative abundance < 1%), such as *Helcococcus*, *Pyramidobacter*, *Christensenellaceae_R-7 group*, *Pseudomonas*, and *Butyrivibrio*, were significantly higher in the LRDS-S and HRDS-S groups (Additional Fig. S5B and C). In addition, Spearman correlation analysis showed that *Bacteroides* and *Acetobacter* were positively associated with pH with significance (rho between 0.57 to 0.59; *P* < 0.05), and *Romboutsia* was positively associated with the concentration of rumen butyrate, valerate, acetate, propionate and total VFAs with significance (rho between 0.58 to 0.77, *P* < 0.05). However, there were weakly correlation between tooth bacteria and the ratio of VFAs (rho between −0.36 to 0.37, *P* > 0.05; Additional Fig. S5D).

### Ruminal and buccal *Prevotellaceae_UCG-003* could serve as a potential discriminative marker for SARA

**Fig. 4:**
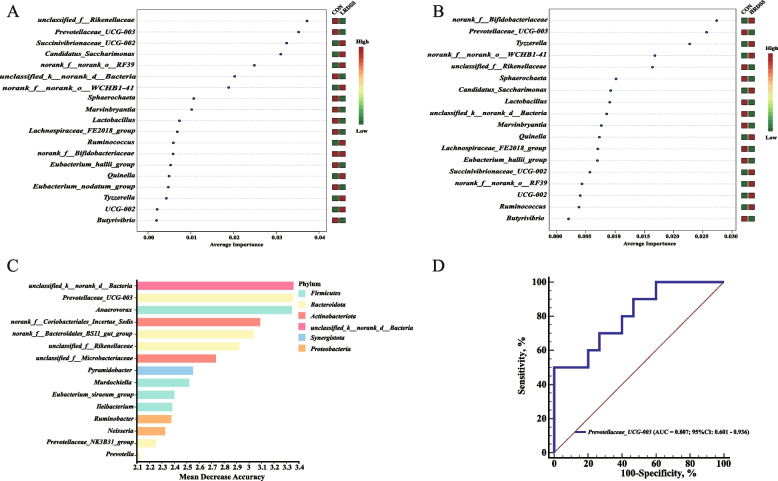
The identification of key ruminal genera to distinguish the goats with SARA occurrence (SARA susceptible) or healthy (control and SARA tolerance) status. **A** and **B** The contribution of each differential ruminal genus to distinguishing the LRDSS group (**A**) or the HRDSS group (**B**) from the CON group was analysed by support vector machine (SVM). **C** The 15 most predictive ruminal genera for classifying samples of the S group versus the H group were selected by random forest classification analysis. **D** The accuracy of distinguishing the S group (SARA occurrence goats, LRDSS and HRDSS groups) from the H group (healthy goats, CON, LRDST and HRDST groups) based on ruminal Prevotellaceae_UCG-003

Further support vector machine (SVM) analyses showed that ruminal *Prevotellaceae_UCG-003* and *unclassified_f__Rikenellaceae* were the two most important genera that distinguished the LRDSS group from the CON group, while *Prevotellaceae_UCG-003* and *norank_f__Bifidobacteriaceae* were the two most important genera that distinguished the HRDSS group from the CON group (Fig. 4A and B). Given that dairy goats in the LRDST and HRDST groups did not develop SARA, we combined the CON, LRDST and HRDST groups into the healthy (H) group and the dairy goats from the LRDSS and HRDSS groups into the SARA (S) group, respectively. Using random forest (RF) classification and receiver operating characteristic (ROC) analysis, *Prevotellaceae_UCG-003* was found among the fifteen most predictive genera to distinguish the S group from the H group with AUC = 0.807 (Fig. 4C).

**Fig. 5:**
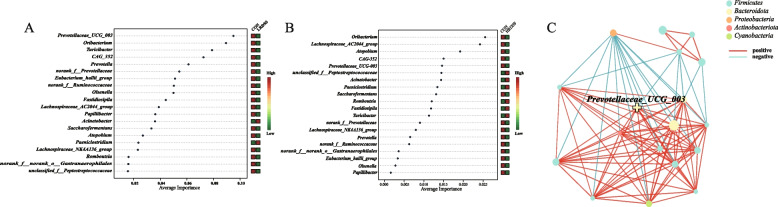
The identification of key buccal genera to distinguish the goats with SARA occurrence (SARA susceptible) or healthy (control and SARA tolerance) status. **A** and **B** The contribution of each buccal differential genus to distinguishing the LRDSS group **(A)** or the HRDSS group (**B**) from the CON group was analysed by a support vector machine (SVM). **C** The correlation among all differential genera based on Spearman's rank correlation coefficient analysis (correlation coefficient > 0.6 and *P* < 0.05). Red and azury lines indicate negative and positive correlations between genera, respectively. Node size reflects relative abundance

Additional SVM analyses revealed that buccal *Prevotellaceae_UCG-003* and *Oribacterium* were the two most important genera distinguishing the LRDSS group from the CON group, and the *Oribacterium* and *Lachnospiraceae_AC2044_group* were the two most important genera distinguishing the HRDSS group from the CON group (Fig. 5A and B). Further, the *Prevotellaceae_UCG-003* was the fifth important genus that distinguished the HRDSS group from the CON group (Fig. 5B). Moreover, the network generated based on buccal genera with significant changes in the LRDSS and HRDSS groups and the degree centrality, closeness centrality, and betweenness centrality of each genus, showed buccal *Prevotellaceae_UCG-003* was the keystone taxon (Fig. 5C; Additional Table S3). Furthermore, *Prevotellaceae_UCG-003* was also found among the fifteen most predictive genera for the classification of the S versus H groups (Additional Fig. S4C). ROC analysis also revealed that the buccal *Prevotellaceae_UCG-003* could distinguish the S group from the H group, with an AUC = 0.779 (Additional Fig. S4D). In comparison, the tooth *Bacteroides* and *Romboutsia* could also help distinguish the S group from the H group, but with a lower AUC (AUC = 0.693 for *Bacteroides* and AUC = 0.593 for *Romboutsia*) (Additional Fig. S5E).

### Relationships between predictive oral bacteria markers related to rumen bacteria-tissue interactions

Further correlation analysis of oral microbiota and rumen microbiota and rumen epithelial gene expression showed that buccal *Prevotellaceae_UCG-003* was positively associated with changes in the rumen *norank_f__norank_o__Bacteroidales* (rho = 0.76; *P* < 0.01), *Marvinbryantia* (rho = 0.67; *P* < 0.01), *Lactobacillus* (rho = 0.82; *P* < 0.01) and *Lachnospiraceae_FE2018_group* (rho = 0.64; *P* < 0.05) and negatively associated with changes in *Tyzzerella* (rho = −0.56; *P* < 0.05) (Additional Fig. S6A). Furthermore, the significantly differential genes enriched in inflammation related pathways were remarkably associated with the abundant of bacteria. Among of them, *SIGLEC15* enriched in regulation of immune system process was positively correlated with *Candidatus_Saccharimonas* (rho = 0.70, *P* < 0.01; Additional Fig. S6B) and *norank_f__Clostridium_methylpentosum_group* (rho = 0.63, *P* < 0.05), repectively. *IL7* enriched in immune system and response related pathways was positively associated with *Ruminobacter* (rho = 0.60, *P* < 0.05), while *IRF7* enriched in immune system and response related pathways was positively related with *Saccharofermentans* (rho = 0.73, *P* < 0.01). On the contrary, *ADCY1* was negatively associated with *norank_f__norank_o__RF39* (rho = −0.55, *P* < 0.05), and CTSL was negatively *Ruminobacter* (rho = −0.62, *P* < 0.05).

### Communications between the rumen and oral microbiota in goats and their relationship with SARA

When the oral microbiota and ruminal microbiota compositions were compared, the microbiota of the rumen and buccal samples had a higher Chao1 index than that of the tooth samples (*P* < 0.01; Additional Fig. S7A). Statistically significant differences in microbial β diversity among the rumen, buccal and tooth samples were also detected (*P* = 0.01; Additional Fig. S7B). The top three dominant genera that were identified in the rumen samples were *Ruminococcus*, *Rikenellaceae_RC9_gut_group* and *Prevotella*. *Corynebacterium*, *Prevotella* and *Streptococcus* were the main genera in the buccal samples, and *Streptococcus*, *Neisseria*, *Veillonella* and *Corynebacterium* were the main genera in the tooth samples (Additional Fig. S7C). Furthermore, the ‘*Ruminococcus* with *Prevotella*’ abundant type was only identified in all rumen samples and some buccal samples of goats, while the ‘*Streptococcus*, *Corynebacterium* and *Neisseria*’ abundant type was identified in some buccal samples and all the tooth samples (Additional Fig. S7D–F).

**Fig. 6:**
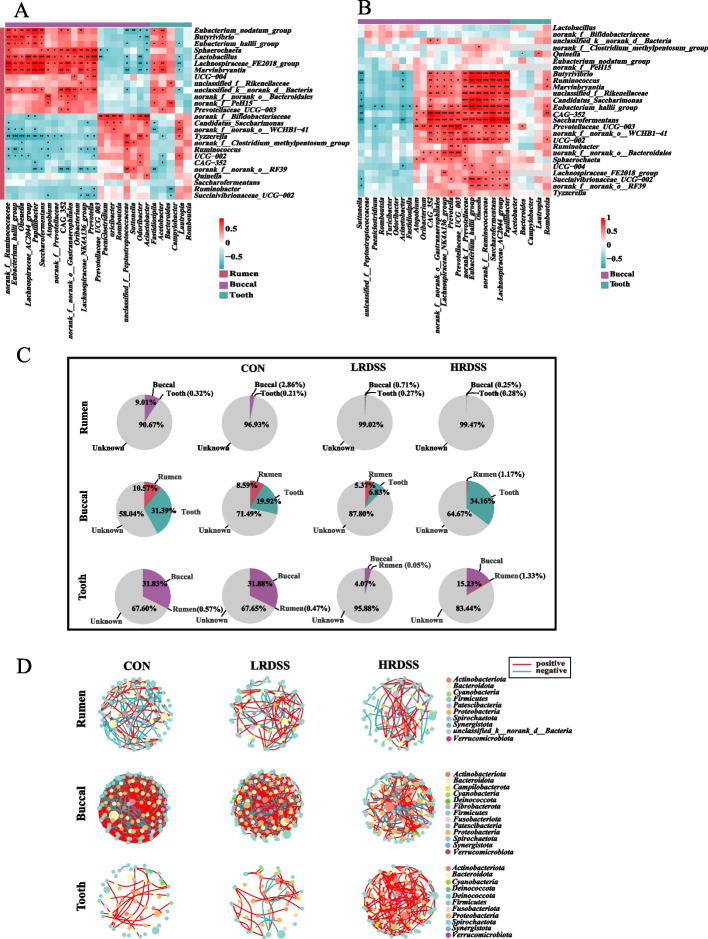
Communications between ruminal and oral (buccal mucosa and tooth) microbiota in dairy goats with or without SARA. **A** Heatmaps of Spearman’s correlation coefficients between the relative abundances of ruminal and oral (buccal mucosa and tooth) microbiota that significantly changed in the LRDSS and HRDSS groups compared with the CON group. **B** Heatmaps of Spearman’s correlation coefficients between SARA-related genera in the oral cavity. **C** SourceTracker analysis to estimate microbial communications from the oral cavity to the rumen and from the rumen to the oral cavity. **D** Microbial abundance cocorrelation networks within ruminal, buccal, or tooth microbiota at the genus level (Based on the genera whose relative abundance larger than 1%). Red and azury lines indicate negative and positive correlations between genera, respectively. Node size reflects relative abundance. * indicates that the difference is significant with *P* < 0.05, ** indicates that the difference is significant with *P* < 0.01, *** indicates that the difference is significant with *P* < 0.001

We then extended our analysis to explore the potential communication between the rumen and oral microbiota in goats with SARA. The correlation analysis of the genera affected by SARA between the rumen and oral sample showed that relative abundance of buccal and tooth genera were significantly higher in the LRDSS and HRDSS groups, and were significantly positively correlated with the rumen *Eubacterium_nodatum_group*, *Butyrivibrio*, *Eubacterium_hallii_group*, *Sphaerochaeta*, *Lactobacillus*, *Lachnospiraceae_FE2018_group*, *Marvinbryantia*, *unclassified_k__norank_d__Bacteria* and *norank_f__norank_o__Bacteroidales* (rho ranged from 0.537 to 0.792; *P* < 0.05; Fig. 6A). When we further inspected these relationships in terms of buccal and tooth sample types, these significant associations remained in the buccal sample type but were lost in the tooth sample type (Fig. 6B). According to source tracker analyses, the proportion of rumen microbiota originating from the buccal mucosa was 9.02%, while the proportion contributed by the tooth-originated microbiota was 0.32%. In terms of the buccal microbiota, 10.57% of them were originated from the rumen with the tooth-originated microbiota constituted a significant portion, accounting for 31.39%. As for the tooth microbiota, 0.57% of them came from the rumen-originated microbiota, whereas 31.83% of them were originated from the buccal mucosa-originated microbiota. With the development of SARA, the proportion of the buccal mucosa-originated microbiota in the rumen gradually decreased, and the proportion of the rumen-originated microbiota in the buccal mucosa also gradually decreased (Fig. 6C). Moreover, microbial networks (at genus level) showed that the SARA-enriched networks had fewer interconnections than CON-enriched networks in the rumen and buccal microbiota (Fig. 6D).

### Ruminal microbiota transplantation (RMT) validated the role of oral and rumen microbiota in SARA

**Fig. 7:**
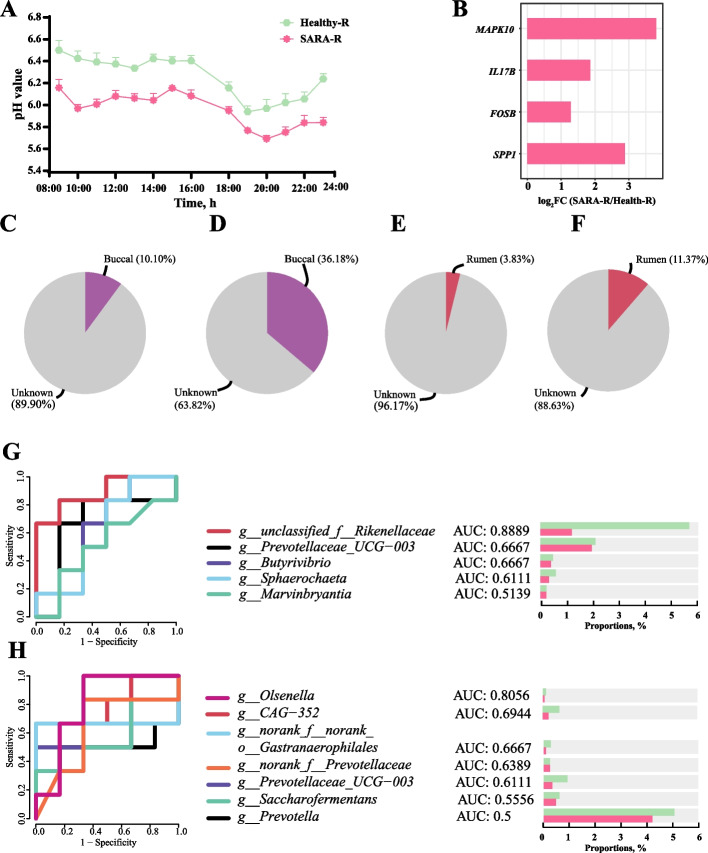
Ruminal microbiota transplantation (RMT) from SARA goats to healthy goats could induce rumen epithelial inflammation and affect the communication between the rumen and oral microbiota of recipient goats. **A** Changes in the rumen pH values (14 h after morning feeding) of 2 groups (Healthy-R and SARA-R) of recipient goats. For the Healthy-R group, the ruminal fluid of the 6 SARA-tolerant (healthy) dairy goats was collected and then transplanted to 6 healthy dairy goats with ruminal fistulas. For the SARA-R group, the ruminal fluid of the 6 donor SARA dairy goats was collected and then transplanted to another 6 healthy dairy goats with ruminal fistulas. **B** Based on the transcriptome study, the ruminal epithelial differential genes with FDR < 0.05 and log_2_fold change > 1 between the Healthy-R and SARA-R groups were involved in the IL-17 signaling pathway, Th17 cell differentiation, and Toll-like receptor signaling pathways. **C **and **D** Source tracker analysis to estimate microbial communications from buccal cavity to rumen in SARA-R group (**C**) and Healthy-R group (**D**). **E** and **F** Source tracker analysis to estimate microbial communications from rumen to buccal cavity in SARA-R group (**E**) and Healthy-R group (**F**). **G** and **H** The significantly changed ruminal (**G**) and buccal (**H**) microbiota genera from the comparison between goats from the Healthy-R and SARA-R groups and the area under the curve (AUC) of receiver operating characteristic (ROC) analyses help to identify the diagnostic accuracy of goats with and without SARA based on the identified significantly changed ruminal (the right part of channel **G**) and buccal (the right part of channel **H**) microbiota genera

After RMT, the rumen fluid pH was significantly decreased in the SARA-R group compared to the Healthy-R group, and the duration of rumen fluid pH less than 5.8 was recorded for more than 3 h in the SARA-R group (Fig. 7A). Furthermore, the expression of genes, such as *MAPK10*, *IL17B*, *FOSB*, and *SPP1*, which mainly participate in the IL-17 signaling pathway, Th17 cell differentiation, and the Toll-like receptor signaling pathway, significantly increased in the SARA-R group (Fig. 7B). After RMT and feeding with a normal concentration of feed for 1 week, no significant differences in α or β diversities of ruminal microbiota were detected between the SARA-R and Healthy-R groups (*P* > 0.05; Additional Fig. S8A–D). Additionally, source-tracker, analysis showed that the buccal mucosa-originated microbiota accounted for 10.10% of the rumen microbiota in the SARA-R group, while the buccal mucosa-originated microbiota accounted for 36.18% of the rumen microbiota in the Healthy-R group (Fig. 7C and D). Moreover, the rumen-originated microbiota accounted for 3.83% of the buccal microbiota in the SARA-R group, and the rumen-originated microbiota accounted for 11.37% of the buccal microbiota in the Healthy-R group (Fig. 7E and F). Further, we performed an ROC analysis of those ruminal and oral differential genera that were previously identified between the SARA (LRDSS and HRDSS groups) and healthy (CON, LRDST and HRDST group) goats, and evaluated by the AUC for distinguishing SARA-R goats from healthy-R goats after RMT were identified (Fig. 7G and H). Of these genera including the oral *Prevotella*, *Prevotellaceae_UCG-003*, *Saccharofermentans*, *CAG-352*, *norank_f_Prevotellaceae*, *norank_f_norank_o_Gastranaerophiales*, *Atopobium*,* Olsenella*,and* Papillibacter*, as well as ruminal *unclassified_f_Rikenellaceae*, *Prevotellaceae_UCG_003*, *Sphaerochaeta*, *Butyrivibrio*, and *Marvinbryantia*, the oral and ruminal *Prevotellaceae_UCG-003*, with AUC numbers of 0.6667 and 0.6389, respectively, to distinguish SARA-R goats from healthy-R goats (Fig. 7G and H).

## Discussion

Our study confirmed that dairy goats have significant individual differences in susceptibility and tolerance to a high-RDS diets as reported in other ruminants [47]. It is noticeable that goats developed SARA even under low level (21.17%) of RDS, while some goats were tolerant under high level (26.72%) RDS. It has been reported that the variation in the expression of the Toll-like receptor genes *TLR2* and *TLR4* in the rumen epithelial wall significantly changed in steers with differential susceptibility to subacute ruminal acidosis [48, 49]. And our rumen epithelial transcriptome analysis showed differential expression of genes related to epithelial inflammation in dairy goats with SARA, but not in the SARA-tolerant goats.

Some previous studies explored the linkage between the individualized ruminal bacteriome and their produced VFAs to varied susceptibilities to SARA [5, 11, 17, 24, 50, 51]. Similar to these studies [5, 11, 17, 24, 50, 51], those starch-degrading bacteria, including the *Ruminococcus, norank_f__norank_o__RF39 and Succinivibrionaceae_UCG-002* [49, 50, 52, 53], were significantly higher in SARA affected goats when compared with the healthy control and SARA tolerant goats. These starch-degrading bacteria have often been reported to take part in the rapid production of VFAs, which leads to a fast drop in ruminal pH [11]. Further, compared with tolerant dairy goats, the significant reduction of fiber-degrading bacteria is a typical feature of rumen microbial communities in SARA dairy goats according to the previous researches [5, 8, 11] and our present study. For instance, previous studies have shown that *Prevotellaceae_UCG-003,* which usually take part in the cellulolytic process [54–57], is extremely sensitive to changes in pH [54, 55, 58], and this taxon was significantly decreased in the rumen of SARA goats. To date, most studies only focused on the shifts in the rumen bacterial community in ruminants with differential SARA susceptibility, the oral bacteria variation between the SARA susceptible and tolerant ruminants are rare, especially in dairy goats.

Compared to previous studies that mainly focused on the rumen microbiota, we also assessed the oral microbiota in dairy goats when they were fed with different levels of RDS. Our study revealed oral microbial composition and its potential interaction with rumen microbiota contributing to the in tolerance and susceptibility of SARA. Previous studies collectively demonstrated the use of both salivary samples and non-invasive buccal swabbing as a method to study the rumen microbiome, and found that buccal swabs are more effectively to represent rumen microbial populations [16, 25, 26, 59]. Similar with these studies, we identified the buccal and tooth swab microbiota in dairy goats, and observed that the buccal swab microbiota shared more taxa with the ruminal bacteria. Only one recent study has identified the varied salivary β-diversity between tolerant and susceptible dairy cows, and found that the salivary genera including the *Bacteroides*, *Desulfovibrio*, *Kandleria*, *Erysipelatoclostridium*, *Coprococcus*, *Ruminobacter*, *Prevotella*, *Lachnospiraceae*, *Ruminococcus gauvreauii_group*, *Alloprevotella*, *Prevotellaceae UCG-003*, and *uncultured Erysipelotrichaceae* could help to classify oral microbiota of SARA-susceptible versus unsusceptible cows [24]. In the present study, we also identified several well-known starch or fiber utilization bacteria, such as *Prevotella*, *Prevotellaceae UCG-003*, *Ruminococcaceae*, *Lachnospiraceae*, *Atopobium*, and *Acinetobacter,* were significantly altered between the buccal samples of healthy and SARA goats and could serve as the buccal bacterial marker. Of these, the *Prevotellaceae UCG-003* was both identified as the significantly altered genus in the previous salivary study [24] and in our present study. Moreover, in the previous study, three shared differential genera including uncultured *Erysipelotrichaceae*, *Prevotella*, and *Anaerovibrio*, of ruminal and salivary samples have been previously identified [24]. Different with this previous study, our findings revealed that the abundances of several significantly changed buccal genera of *Prevotella* and *Prevotellaceae_UCG-003*, which have been documented as cellulolytic genera [54, 60], showed significant increased trends in healthy goats and could effectively differentiate SARA from healthy dairy goats. To sum up, these microbial results indicated that variations in SARA susceptibility might be mirrored within the bacterial community of buccal samples, implying that the buccal microbiota is potentially associated with alterations in the rumen microbiota.

Considering the strong interaction between oral and gastrointestinal microbiota [22, 52], we further explored the relationship between oral and rumen microbiota in dairy goats. Oral microbiota, especially the bacteria in the buccal cavity, were significantly correlated with the rumen microbiota. Several previous studies have shown that the oral microbiota can also reflect fluctuations in the rumen microbiota in a timely manner when oral and rumen samples were compared under different feeding strategies (high or low grain feeding) or at different ages and durations of weaning [16, 24–26]. This suggests that there may be a connection between these two microbial communities. The additional source tracker analyses suggested a decline in transmission between the rumen and buccal microbiota in SARA-susceptible dairy goats compared to those SARA-tolerant ones. Microbial network analysis at the genus level revealed that the microbial connections between the rumen and buccal cavity gradually decreased with the development of SARA. Moreover, the introduction of rumen contents of SARA-susceptible goats to healthy goats using RMT confirmed that the ruminal microbiota of SARA-susceptible goats triggered a decrease in the transmission between the rumen and buccal microbiota of recipient goats. These findings suggest that the rumen microflora may affect the buccal microbiota or vice versa and have provided further evidence of possible communication between the rumen and buccal microbiota.

Ruminal pH is an important index for SARA discrimination [53, 58], and when it was used as an environmental factor in the interaction analysis with buccal microbiota, we found that changes in pH were associated with buccal microflora variation. There was a significant positive correlation between ruminal pH and the abundance of buccal *Prevotella, Prevotellaceae*_*UCG-003, norank_f__Prevotellaceae, Lachnospiraceae*_*AC2044_group* and *Lachnospiraceae*_*NK4A136_group*, which have been reported to be cellulolytic genera [54–56]. According to the previous studies, these cellulolytic genera were prone to lysis at low pH condition in the rumen [8, 11]. Hence, the decreased abundance of these cellulolytic and pH sensitive genera in buccal samples again suggests the communication of ruminal-oral microbiota, especially the microbial migration from the rumen to the oral cavity, could be the key factors that affect the buccal bacterial communities. The most noticeable bacteria with reduced abundance in SARA-susceptible goats was *Prevotellaceae*_*UCG-003* in the rumen, which is known as a cellulolytic bacterium [54–57]. SVM and random forest classification analyses showed that ruminal *Prevotellaceae*_*UCG-003* distinguished SARA-susceptible goats from healthy goats, including SARA-tolerant goats. The sensitivity of *Prevotellaceae*_*UCG-003* to changes in pH has been well-documented [54, 60, 61], suggesting that *Prevotellaceae*_*UCG-003* can serve as a valuable tool for identifying the onset of SARA. In the buccal samples, *Prevotellaceae_UCG-003* as the bacteria with the most remarkable differences between groups was noticed [54, 60, 61], suggesting that buccal *Prevotellaceae*_*UCG-003* was the key microbial indicator for the occurrence of SARA as well. Further bacterial interaction network revealed that *Prevotellaceae*_*UCG-003* was the core genus of these differential bacteria, and we verified that *Prevotellaceae*_*UCG-003* in the buccal cavity may serve as the basis for determining the occurrence of SARA. These suggest that when SARA occurred, the abundance of oral *Prevotellaceae*_*UCG-003* was affected and reduced and the interaction between the rumen and oral microbiota could disrupted. Furthermore, RMT from SARA-susceptible and SARA-tolerant goats to healthy goats confirmed that buccal *Prevotellaceae_UCG-003* could also serve as a potential microbial for diagnosing SARA after RMT. Moreover, we established the correlation among oral and rumen microbiota and rumen epithelial immune-related genes expression, which suggest that buccal and rumen microbiota interaction may affect expression of genes involved in ruminal inflammation when SARA occurrence. However, it should be noted that due to sequencing methods, the *Prevotellaceae_UCG-003* we identified was not a specific bacterium, but a genus with a similar genetic characteristic. The specific species of *Prevotellaceae_UCG-003* need to be further selected and these relationships need to be further verified and the causal effects needs to be better determined in future studies. In addition, it is important to acknowledge the limitations of this study, particularly due to the small sample size. However, given that the fundamental principles of rumination and rumen function are conserved across ruminant species, our findings suggest that oral microbiota can serve as an indicator of SARA occurrence and rumen health and may function as a diagnostic tool. These results can inform hypotheses and experimental designs for future studies in larger ruminants.

## Conclusion

The results of this study revealed SARA led to dysbiosis of the rumen and oral microbiota, which can be affected through rumination and communication between the rumen and oral microbiota, and identified oral *Prevotellaceae*_*UCG-003* as a potential microbial marker to diagnosis SARA. It is noticeable that although significant differences in rumen and oral microflora between the dairy goat groups were identified, this was a small cohort of dairy goats, which need to be validated in a larger population and multiple farms. Although limited by the use of 16S rRNA gene sequencing, it is hard to reflect the actual changes in the microbiome and microbial function. The 16S rRNA gene sequencing, which is a more economical approach, can provide more accurate results when detecting the microflora composition of samples (such as the buccal samples) with low bacterial abundance and high host contamination [62]. Additionally, the identified association among rumen bacteria, buccal bacteria and the functions of rumen epithelial genes could provide new microbial insights into potential microbial marker for SARA in dairy goats. Taken together, our findings of oral microbiota and its connection with rumen microbiota can lead to novel strategies for potential diagnosis of SARA.

## Supplementary Information

Additional file 1: Fig. S1 Analysis of gene expression in the rumen epithelium. (A) Venn diagram of the identified genes in the CON, LRDSS and HRDSS groups. (B) The numbers of differentially expressed genes (DEGs) in CON vs. LRDSS and LRDST vs. LRDSS. Red and blue indicate upregulated and downregulated genes, respectively. Fig. S2 Ruminal differential genera identified in the comparison among groups of dairy goats varying between SARA susceptibility. (A) The number of ASVs in each rumen samples. (B) The number of filtered sequences in each rumen samples. (C and D) Comparison of ruminal microbial alpha diversity with the Chao1 index (C) and PD index (D) among the CON, LRDSS, LRDST, HRDSS, and HRDST groups. (E) Differential genera selected from the comparison between the LRDST group and the LRDSS group. (F) Differential genera selected from the comparison between the HRDST group and the HRDSS group. * indicates that the difference is significant at *P* < 0.05, ** indicates that the difference is significant at *P* < 0.01, *** indicates that the difference is significant at *P* < 0.001. Fig. S3 The numbers of ASVs and sequences in buccal cavity and tooth. (A-B) The number of ASVs (A) and filtered sequences (B) in each buccal sample. (C and D) The number of ASVs (C) and filtered sequences (D) in each tooth sample. Fig. S4 Buccal differential genera identified in the comparison among groups of dairy goats varying in SARA susceptibility. (A) Differential genera selected from the comparison between the LRDST group and the LRDSS group. (B) Differential genera selected from the comparison between the HRDST group and the HRDSS group. * indicates that the difference is significant at *P* < 0.05, ** indicates that the difference is significant at *P* < 0.01, *** indicates that the difference is significant at *P*< 0.001. (C) The 15 most predictive buccal genera to classify samples of the S group versus the H group were selected by random forest classification analysis. (D) The accuracy of distinguishing the S group from the H group based on buccal *Prevotellaceae_UCG-003*. Fig. S5 Comparison of tooth microbiota of dairy goats exhibiting SARA occurrence (SARA susceptible) or healthy (control and SARA tolerance) status. (A) The differential genera identified when the comparison between the CON group and SARA (LRDSS and HRDSS) groups was performed. (The genera that gradually increased along the CON, LRDSS and HRDSS groups are highlighted in brown, and the genera that gradually decreased along the CON group, LRDSS group and HRDSS group are highlighted in purple). (B) Differential genera selected from the comparison between the LRDST group and the LRDSS group. (C) Differential genera selected from the comparison between the HRDST group and the HRDSS group. * indicates that the difference is significant with FDR < 0.05, ** indicates that the difference is significant with FDR < 0.01, *** indicates that the difference is significant with FDR < 0.001. (D) Spearman correlation between the common genus-level differences in the bacteria in dairy goats from the CON group and SARA (LRDSS and HRDSS) groups and their rumen fermentation parameters. (E) The accuracy of distinguishing SARA dairy goats from healthy dairy goats based on tooth Bacteroides and Romboutsia. The figures presented from left to right were based on the comparison groups of the CON vs. LRDSS, CON vs. HRDSS, and health vs. SARA. Fig. S6 The identification of the association between oral microbiota and rumen microbiota and the connection between rumen microbiota and genes that affected the occurrence of epithelial inflammation. (A) The correlation among buccal Prevotellaceae_UCG-003, ruminal differential genera and the genes that were differentially expressed in ruminal epithelium based on Spearman's rank correlation coefficient analysis (correlation coefficient > 0.6 and *P* < 0.05). (B) The affiliation relationship between identified rumen epithelial immune-related differentially expressed genes and their involved GO enrichment terms, the genes showed in this graph were all significantly associated with the identified differential ruminal bacteria. Fig. S7 Comparison of ruminal and oral microbiota. (A) Chao1 index of ruminal and oral (buccal mucosa and tooth) microbiota. * indicates that the difference is significant at *P* < 0.05, ** indicates that the difference is significant at *P* < 0.01, *** indicates that the difference is significant at *P* < 0.001. (B) Principal coordinate analysis (PCoA) of ruminal and oral (buccal mucosa and tooth) microbiota. (C) Average relative abundance of microbiota at the genus level of ruminal and oral (buccal mucosa and tooth) microbiota; those bacteria whose relative abundance was less than 1% were classified as others. (D) Two different microbial types were identified based on the genera of ruminal and oral (buccal mucosa and tooth) sample types. (E) The microbial type distribution was compared between ruminal, buccal and tooth sample types. (F) Average relative abundance of microbiota at the genus level of the two different microbial types; those bacteria whose relative abundance was less than 1% were classified as others. Fig. S8 The oral and ruminal microbiota diversity comparison between the Healthy-R and SARA-R groups. (A-B) The oral Chao1 index (A) and beta diversity (B) were compared between goats from the Healthy-R and SARA-R groups. (C-D) The ruminal Chao1 index (C) and beta diversity (D) were compared between goats from the Healthy-R and SARA-R groups. The Mann-Whitney U test was employed to test microbial alpha diversity differences between the two groups. ANOSIM analysis based on Bray-Curtis distance matrices was used to identify beta diversity differences. Table S1. The ingredients and nutrient composition of the three diets on a dry matter (DM) basis. Table S2. Comparison of rumen LPS and lactate concentrations among the CON, LRDST, LRDSS, HRDST and HRDSS groups. Table S3. Identification of key genera based on calculated degree centrality, closeness centrality, and betweenness centrality.

## Data Availability

All the data generated or analysed for this study are included in this paper. The 16S rRNA gene sequences are available in the Sequence Read Archive (SRA) of NCBI under accession project number PRJNA941439. The RNA-seq data were deposited into the China National Center for Bioinformation (CNCB; https://www.cncb.ac.cn/?lang=en) with accession number CRA010208.

## Abbreviations

AUC: Area under the curve
CCA: Canonical correspondence analysis
DCA: Detrended correspondence analysis
DEG: Differentially expressed gene
DM: Dry matter
FDR: False discovery rate
FID: Flame ionization detector
LPS: Lipopolysaccharide
PBS: Phosphate-buffered saline
PCoA: Principal coordinate analysis
RDA: Redundancy analysis
RF: Random forest
RMT: Ruminal microbiota transplantation
ROC: Receiver operating characteristic
SARA: Subacute rumen acidosis
SVM: Support vector machine
TMR: Total mixed ration
VFA: Volatile fatty acid
