# Supplementary material for: Ruminal-buccal microbiota transmission and their diagnostic roles in subacute rumen acidosis in dairy goats
Source: J Anim Sci Biotechnol. 2025 Mar 2;16:32. doi: 10.1186/s40104-025-01162-4 (PMC11872310; doi:10.1186/s40104-025-01162-4)
Supplement: Supplementary file 1 — Additional file 1: Fig. S1 Analysis of gene expression in the rumen epithelium. (A) Venn diagram of the identified genes in the CON, LRDSS and HRDSS groups. (B) The numbers of differentially expressed genes (DEGs) in CON vs. LRDSS and LRDST vs. LRDSS. Red and blue indicate upregulated and downregulated genes, respectively. Fig. S2 Ruminal differential genera identified in the comparison among groups of dairy goats varying between SARA susceptibility. (A) The number of ASVs in each rumen samples. (B) The number of filtered sequences in each rumen samples. (C and D) Comparison of ruminal microbial alpha diversity with the Chao1 index (C) and PD index (D) among the CON, LRDSS, LRDST, HRDSS, and HRDST groups. (E) Differential genera selected from the comparison between the LRDST group and the LRDSS group. (F) Differential genera selected from the comparison between the HRDST group and the HRDSS group. * indicates that the difference is significant at P < 0.05, ** indicates that the difference is significant at P < 0.01, *** indicates that the difference is significant at P < 0.001. Fig. S3 The numbers of ASVs and sequences in buccal cavity and tooth. (A-B) The number of ASVs (A) and filtered sequences (B) in each buccal sample. (C and D) The number of ASVs (C) and filtered sequences (D) in each tooth sample. Fig. S4 Buccal differential genera identified in the comparison among groups of dairy goats varying in SARA susceptibility. (A) Differential genera selected from the comparison between the LRDST group and the LRDSS group. (B) Differential genera selected from the comparison between the HRDST group and the HRDSS group. * indicates that the difference is significant at P < 0.05, ** indicates that the difference is significant at P < 0.01, *** indicates that the difference is significant at P< 0.001. (C) The 15 most predictive buccal genera to classify samples of the S group versus the H group were selected by random forest classification analysis. (D) The accuracy of [file 40104_2025_1162_MOESM1_ESM.pdf]

Supplementary Information

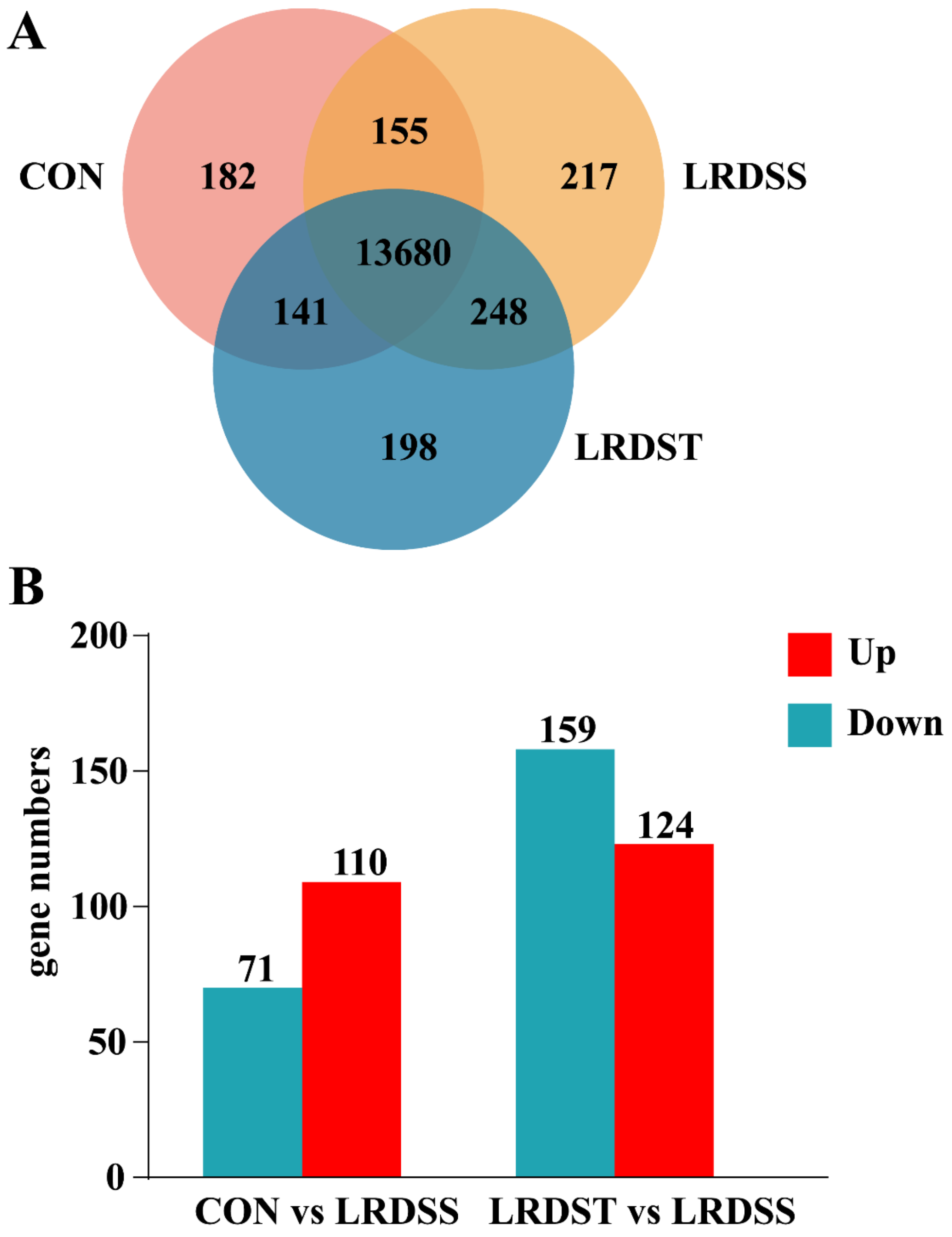

Fig. S1 Analysis of gene expression in the rumen epithelium. (**A)** Venn diagram of the identified genes in the CON, LRDSS and HRDSS groups. (**B)** The numbers of differentially expressed genes (DEGs) in CON vs LRDSS and LRDST vs LRDSS. Red and blue indicate upregulated and downregulated genes, respectively.

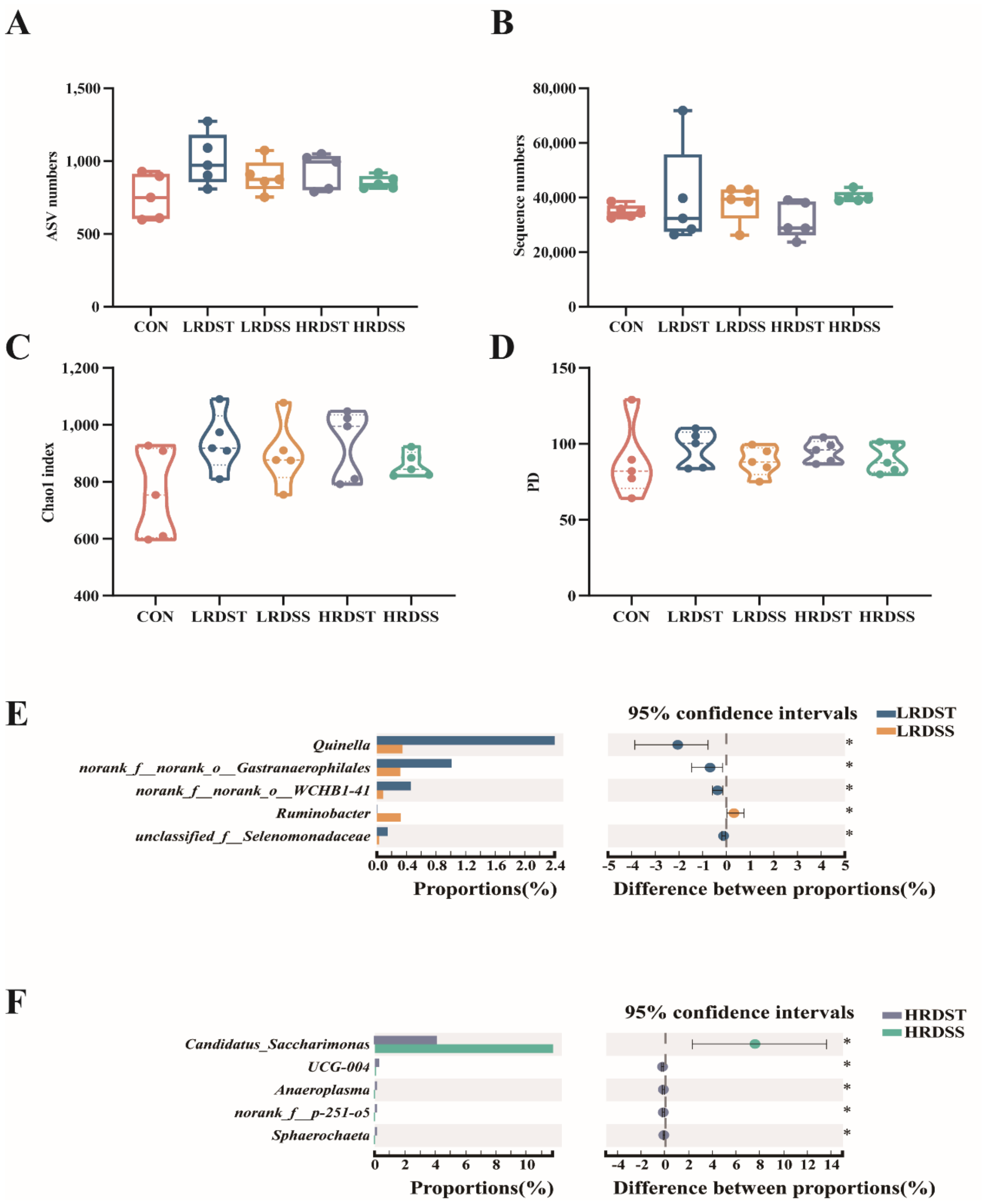

Fig. S2 Ruminal differential genera identified in the comparison among groups of dairy goats varying between SARA susceptibility. **(A)** The number of ASVs in each rumen samples. **(B)** The number of filtered sequences in each rumen samples. **(C-D)** Comparison of ruminal microbial alpha diversity with the Chao1 index **(C)** and PD index **(D)** among the CON, LRDSS, LRDST, HRDSS, and HRDST groups. **(E)** Differential genera selected from the comparison between the LRDST group and the

LRDSS group. **(F)** Differential genera selected from the comparison between the HRDST group and the HRDSS group. * indicates that the difference is significant at $P < 0.05$, ** indicates that the difference is significant at $P < 0.01$, *** indicates that the difference is significant at $P < 0.001$.

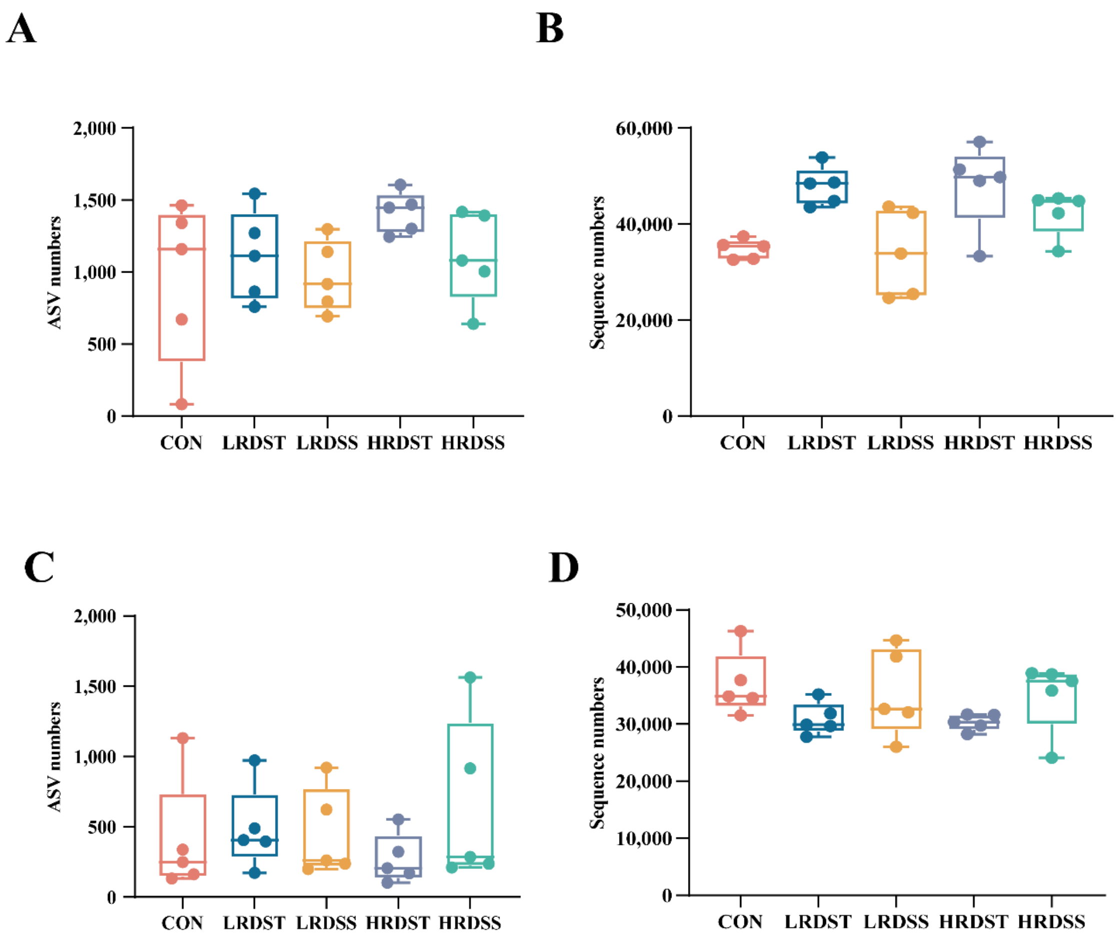

Fig. S3 The numbers of ASVs and sequences in buccal cavity and tooth. **(A-B)** The number of ASVs **(A)** and filtered sequences **(B)** in each buccal sample. **(C-D)** The number of ASVs **(C)** and filtered sequences **(D)** in each tooth sample.

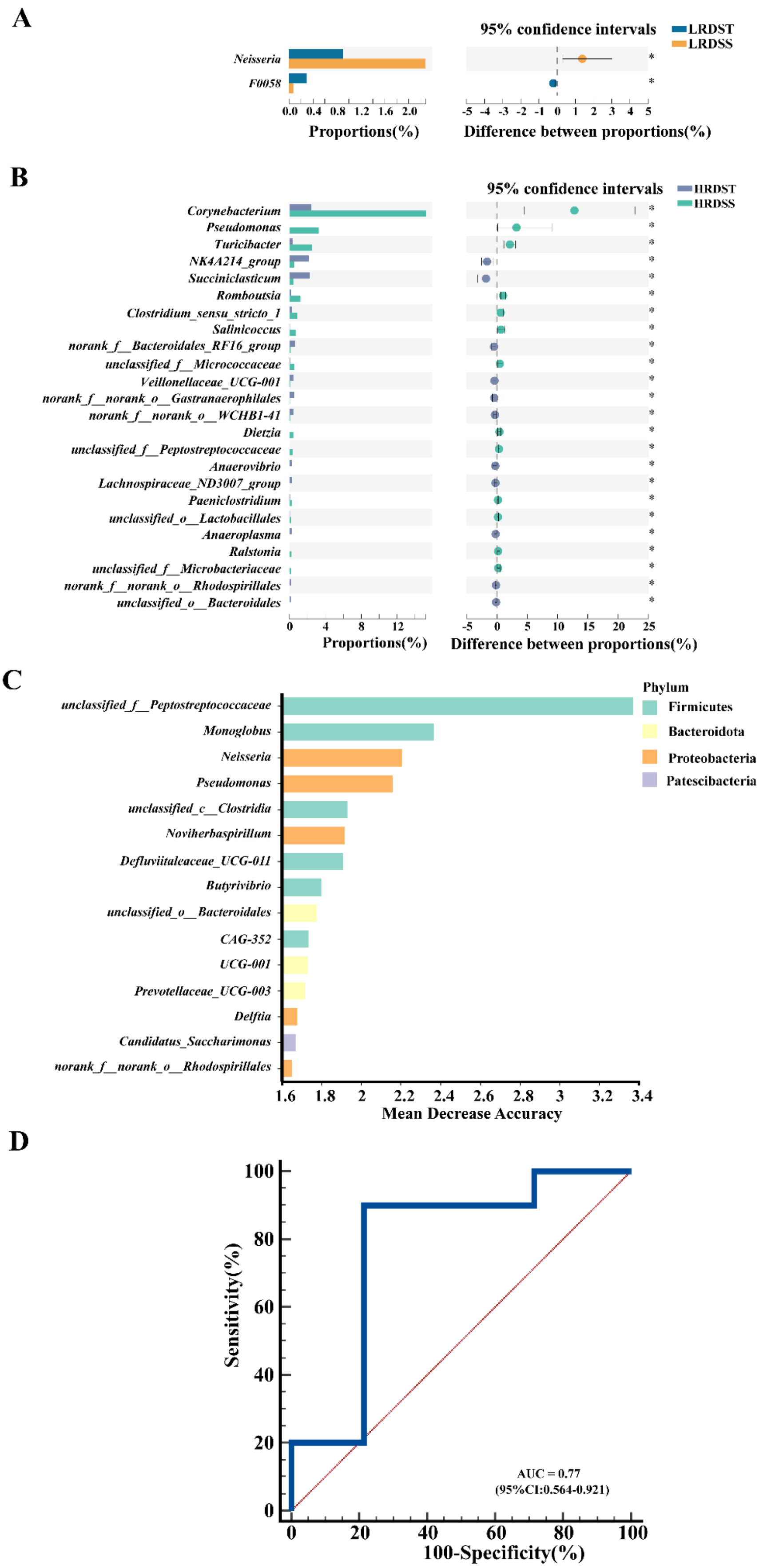
A
95% confidence intervals
LRDST
LRDSS
Neisseria
F0058
0.0 0.4 0.8 1.2 1.6 2.0
Proportions(%)
-5 -4 -3 -2 -1 0 1 2 3 4 5
Difference between proportions(%)
B
95% confidence intervals
HRDST
HRDSS
Corynebacterium
Pseudomonas
Turicibacter
NK4A214_group
Succiniclasticum
Romboutsia
Clostridium_sensu_stricto_1
Salinicoccus
norank_f__Bacteroidales_RF16_group
unclassified_f__Micrococcaceae
Veillonellaceae_UCG-001
norank_f__norank_o__Gastranaerophilales
norank_f__norank_o__WCHB1-41
Dietzia
unclassified_f__Peptostreptococcaceae
Anaerovibrio
Lachnospiraceae_ND3007_group
Paeniclostridium
unclassified_o__Lactobacillales
Anaeroplasma
Ralstonia
unclassified_f__Microbacteriaceae
norank_f__norank_o__Rhodospirillales
unclassified_o__Bacteroidales
0 4 8 12
Proportions(%)
-5 0 5 10 15 20 25
Difference between proportions(%)
C
Phylum
Firmicutes
Bacteroidota
Proteobacteria
Patescibacteria
unclassified_f__Peptostreptococcaceae
Monoglobus
Neisseria
Pseudomonas
unclassified_c__Clostridia
Noviherbaspirillum
Defluviitaleaceae_UCG-011
Butyrivibrio
unclassified_o__Bacteroidales
CAG-352
UCG-001
Prevotellaceae_UCG-003
Delftia
Candidatus_Saccharimonas
norank_f__norank_o__Rhodospirillales
1.6 1.8 2 2.2 2.4 2.6 2.8 3 3.2 3.4
Mean Decrease Accuracy
D
Sensitivity(%)
0 20 40 60 80 100
100-Specificity(%)
0 20 40 60 80 100
AUC = 0.77
(95%CI:0.564-0.921)

Fig. S4 Buccal differential genera identified in the comparison among groups of dairy goats varying in SARA susceptibility. (**A)** Differential genera selected from the comparison between the LRDST group and the LRDSS group. (**B)** Differential genera selected from the comparison between the HRDST group and the HRDSS group. * indicates that the difference is significant at $P < 0.05$, ** indicates that the difference is significant at $P < 0.01$, *** indicates that the difference is significant at $P < 0.001$. **(C)** The 15 most predictive buccal genera to classify samples of the S group versus the H group were selected by random forest classification analysis. (**D)** The accuracy of distinguishing the S group from the H group based on buccal *Prevotellaceae_UCG-003*.

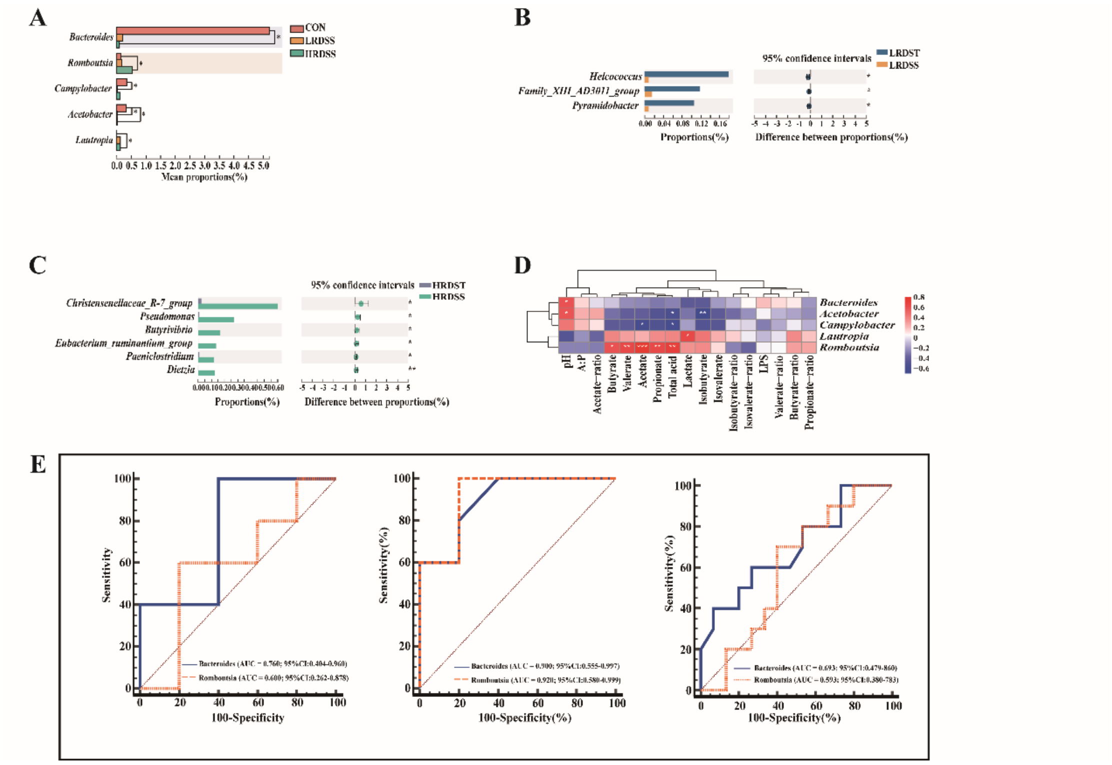

Fig. S5 Comparison of tooth microbiota of dairy goats exhibiting SARA occurrence (SARA susceptible) or healthy (control and SARA tolerance) status. (**A**) The differential genera identified when the comparison between the CON group and SARA (LRDSS and HRDSS) groups was performed. (The genera that gradually increased along the CON, LRDSS and HRDSS groups are highlighted in brown, and the genera that gradually decreased along the CON group, LRDSS group and HRDSS group are highlighted in purple). (**B**) Differential genera selected from the comparison between the LRDST group and the LRDSS group. **(C)** Differential genera selected from the comparison between the HRDST group and the HRDSS group. * indicates that the difference is significant with FDR < 0.05, ** indicates that the difference is significant with FDR < 0.01, *** indicates that the difference is significant with FDR < 0.001. (**D**) Spearman correlation between the common genus-level differences in the bacteria in dairy goats from the CON group and SARA (LRDSS and HRDSS) groups and their

rumen fermentation parameters. (**E)** The accuracy of distinguishing SARA dairy goats from healthy dairy goats based on tooth Bacteroides and Romboutsia. The figures presented from left to right were based on the comparison groups of the CON vs LRDSS, CON vs HRDSS, and health vs SARA.

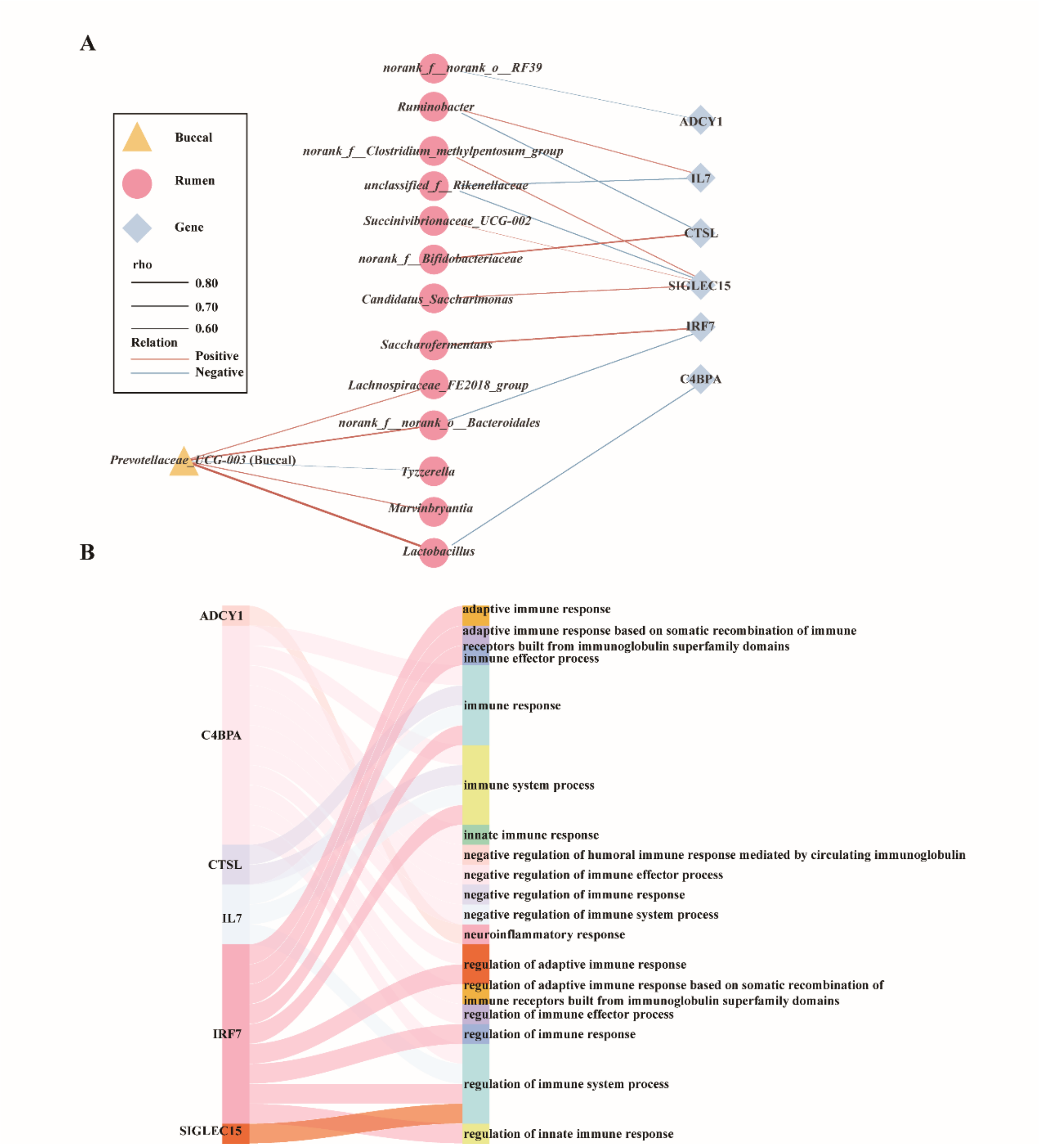

Fig. S6 The identification of the association between oral microbiota and rumen microbiota and the connection between rumen microbiota and genes that affected the occurrence of epithelial inflammation. **(A)** The correlation among buccal *Prevotellaceae_UCG-003*, ruminal differential genera and the genes that were differentially expressed in ruminal epithelium based on Spearman's rank correlation coefficient analysis (correlation coefficient > 0.6 and $P < 0.05$). **(B)** The affiliation relationship between identified rumen epithelial immune-related differentially

expressed genes and their involved GO enrichment terms, the genes showed in this graph were all significantly associated with the identified differential ruminal bacteria.

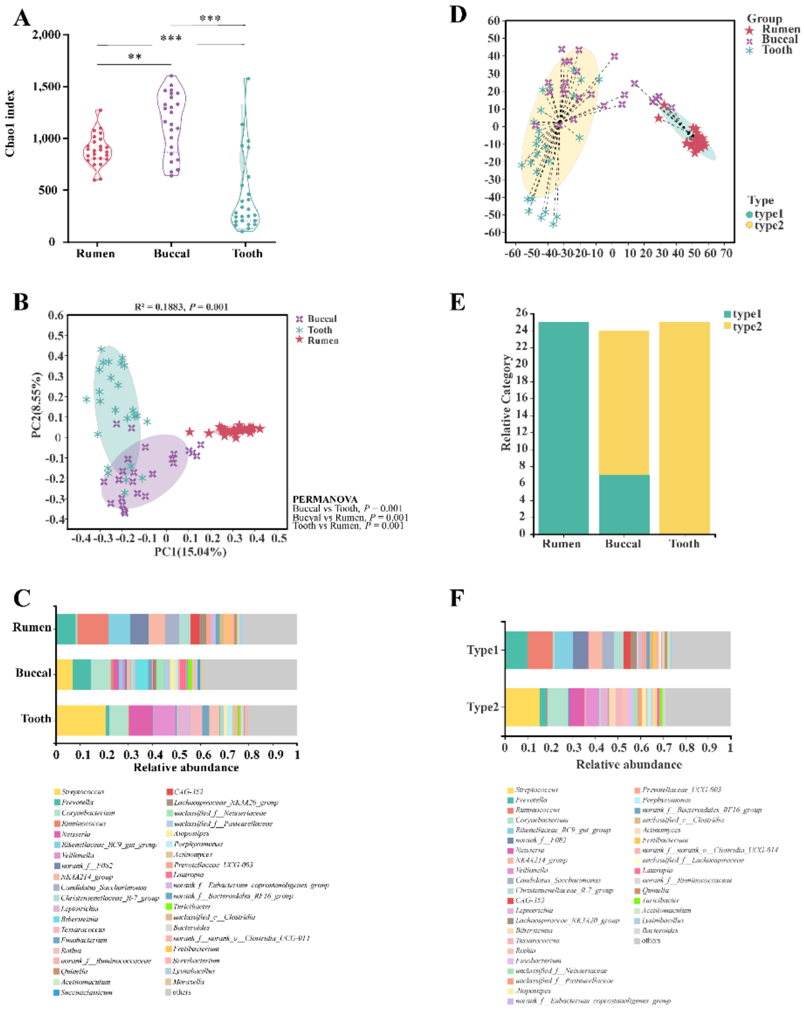

Fig. S7 Comparison of ruminal and oral microbiota. **(A)** Chao1 index of ruminal and oral (buccal mucosa and tooth) microbiota. * indicates that the difference is significant at $P < 0.05$, ** indicates that the difference is significant at $P < 0.01$, *** indicates that the difference is significant at $P < 0.001$. **(B)** Principal coordinate analysis (PCoA) of ruminal and oral (buccal mucosa and tooth) microbiota. **(C)** Average relative abundance of microbiota at the genus level of ruminal and oral (buccal mucosa and tooth) microbiota; those bacteria whose relative abundance was less than 1% were classified as others. **(D)** Two different microbial types were identified based on the genera of ruminal and oral (buccal mucosa and tooth) sample types. **(E)** The microbial type

distribution was compared between ruminal, buccal and tooth sample types. **(F)** Average relative abundance of microbiota at the genus level of the two different microbial types; those bacteria whose relative abundance was less than 1% were classified as others.

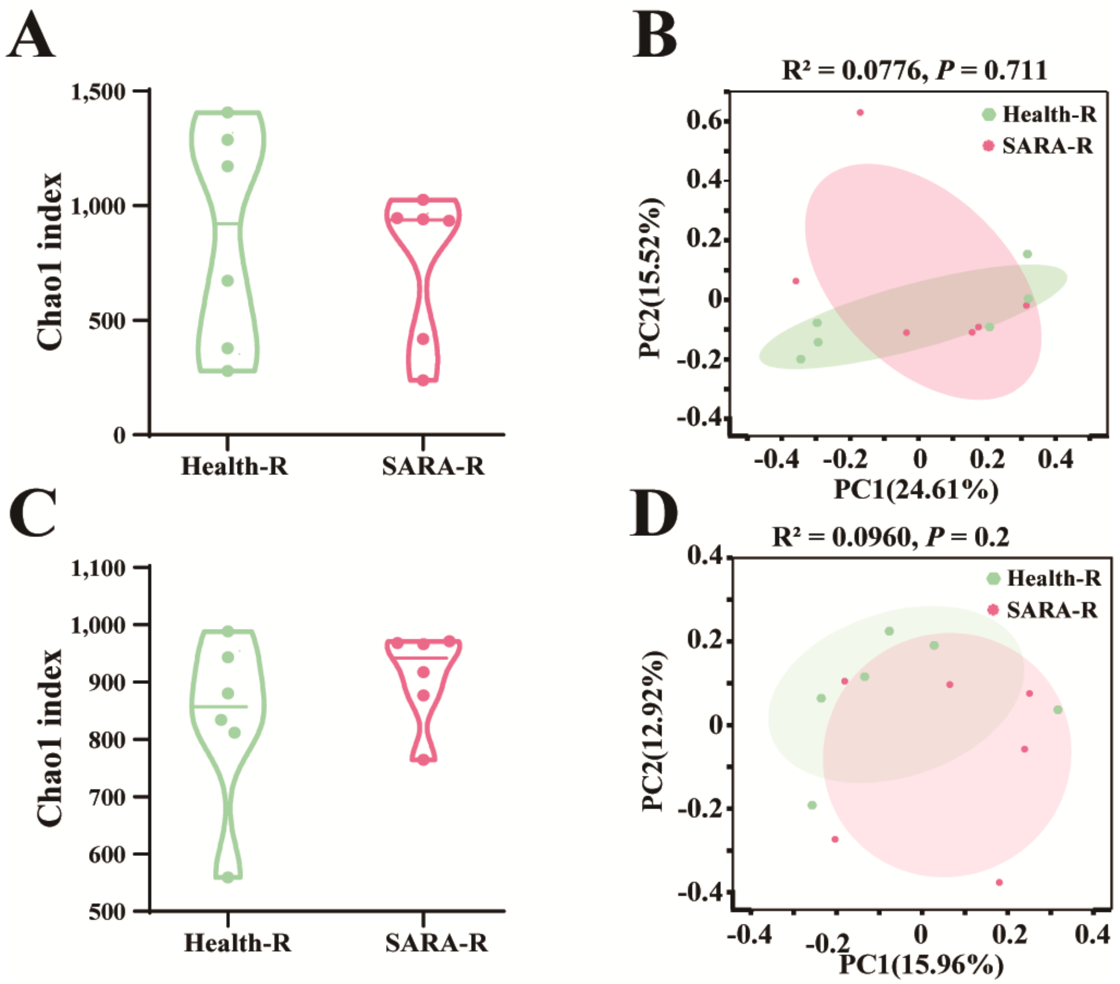

Fig. S8 The oral and ruminal microbiota diversity comparison between the Healthy-R and SARA-R groups. (**A-B)** The oral Chao1 index **(A)** and beta diversity **(B)** were compared between goats from the Healthy-R and SARA-R groups. **(C-D)** The ruminal Chao1 index **(C)** and beta diversity **(D)** were compared between goats from the Healthy-R and SARA-R groups. The Mann–Whitney U test was employed to test microbial alpha diversity differences between the two groups. ANOSIM analysis based on Bray-Curtis distance matrices was used to identify beta diversity differences.

Table S1. The ingredients and nutrient composition of the three diets on a dry matter (DM) basis.

Item	Treatments		
	CON	LRDS	HRDS
Ingredient (%of DM)			
Corn silage	46.20	19.80	19.80
Alfalfa hay	23.80	10.20	10.20
Whole corn	17.53	40.90	0.00
Gorund corn	0.00	0.00	40.90
Wheat bran	8.65	20.19	20.19
Soybean meal	2.26	5.27	5.27
Limestone	0.57	2.33	2.33
Salt	0.09	0.22	0.22
Vitamin-mineral mix[1]	0.90	2.10	2.10

Nutrient composition			
DM[2] (%)	46.69	64.07	64.07
NE_L (MJ/Kg)	4.02	5.53	6.24
CP (%)	10.42	12.38	12.38
Starch (%)	16.91	35.96	36.12
NFC (%)	30.94	45.37	45.53
NDF (%)	49.22	31.99	32.00
ADF (%)	31.58	17.77	17.77

[1]Vitamin-mineral mix (per kilogram): 450 mg of nicotinic acid, 600 mg of Mn, 950 mg of Zn, 430 mg of Fe, 650 mg of Cu, 30 mg of Se, 45 mg of I, 20 mg of Co, 800 mg of vitamin E, 45,000 IU of vitamin D, and 120,000 IU of vitamin A.

[2]DM, dry matter; NE_L, net energy of lactation; CP, crude protein; NFC, non-fiber carbohydrate; NDF, neutral detergent fibre and ADF, acid detergent fibre.

Table S2. Comparison of rumen LPS and lactate concentrations among the CON, LRDST, LRDSS, HRDST and HRDSS groups. [a-c] Mean values within an index with the same superscript letters indicated no significant difference (P < 0.05).

Item	Group					SEM	*P*-value
	CON	LRDST	LRDSS	HRDST	HRDSS		
VFA concdntration (mmol/L)							
Acetate	56.51	57.53	69.82	61.41	75.00	2.54	0.073
Propionate	18.54b	18.94b	25.05ab	21.26ab	28.44a	1.28	0.048
Isobutyrate	0.79b	1.00ab	1.23a	1.07a	1.15a	0.04	0.005
Butyrate	10.23	11.19	13.86	11.69	16.53	0.79	0.069
Isovalerate	1.21b	1.45ab	1.84a	1.37b	1.55ab	0.07	0.045
Valerate	1.51	1.73	2.17	1.75	2.28	0.11	0.124
Total acid	88.79b	91.83b	113.97ab	98.55ab	124.95a	4.60	0.044
VFA proportion (%)							

Acetate	63.94	63.09	61.55	62.62	60.00	0.64	0.348
Propionate	20.53	20.40	21.85	21.01	22.78	0.41	0.339
Isobutyrate	0.91	1.10	1.08	1.20	0.92	0.05	0.357
Butyrate	11.52	11.95	12.03	11.82	13.22	0.40	0.739
Isovalerate	1.41	1.59	1.63	1.57	1.25	0.09	0.694
Valerate	1.69	1.87	1.86	1.78	1.83	0.04	0.713
Acetate:Propionate	3.18	3.12	2.83	3.03	2.64	0.09	0.299
Lactate (mmol/L)	0.52c	0.82bc	0.63bc	0.93ab	1.20a	0.06	<0.001
LPS (EU/ml)	6182.67	4743.18	9250.33	7679.40	6394.85	636.93	0.225

Table S3. Identification of key genera based on calculated degree centrality, closeness centrality, and betweenness centrality.

Node_ID	Node_Name	Degree_Centrality	Closeness_Centrality	Betweenness_Centrality
1	*g__Turicibacter*	0.176470588	0.435897436	0

2	*g__Romboutsia*	0.294117647	0.586206897	0.045343137
3	*g__Prevotellaceae_UCG-003*	0.823529412	0.85	0.027751783
4	*g__Lachnospiraceae_AC2044_group*	0.764705882	0.739130435	0.002016488
5	*g__CAG-352*	0.764705882	0.739130435	0.002016488
6	*g__unclassified_f__Peptostreptococcaceae*	0.176470588	0.435897436	0
7	*g__Lachnospiraceae_NK4A136_group*	0.647058824	0.68	0
8	*g__norank_f__Prevotellaceae*	0.882352941	0.894736842	0.083511586
9	*g__Acinetobacter*	0.764705882	0.80952381	0.022058824
10	*g__Olsenella*	0.764705882	0.739130435	0.002016488
11	*g__Prevotella*	0.823529412	0.85	0.027751783
12	*g__norank_f__norank_o__Gastranaerophilales*	0.764705882	0.739130435	0.002016488
13	*g__norank_f__Ruminococcaceae*	0.764705882	0.739130435	0.002016488
14	*g__Eubacterium_hallii_group*	0.823529412	0.85	0.078487077

15	*g__Saccharofermentans*	0.764705882	0.739130435	0.002016488
16	*g__Papillibacter*	0.764705882	0.739130435	0.002016488
17	*g__Paeniclostridium*	0.529411765	0.68	0.172181373
18	*g__Oribacterium*	0.705882353	0.772727273	0.021446078
